# *In Situ* Diazotroph Population Dynamics Under Different Resource Ratios in the North Pacific Subtropical Gyre

**DOI:** 10.3389/fmicb.2018.01616

**Published:** 2018-07-25

**Authors:** Kendra A. Turk-Kubo, Paige Connell, David Caron, Mary E. Hogan, Hanna M. Farnelid, Jonathan P. Zehr

**Affiliations:** ^1^Department of Ocean Sciences, University of California, Santa Cruz, Santa Cruz, CA, United States; ^2^Department of Biological Sciences, University of Southern California, Los Angeles, CA, United States; ^3^Centre for Ecology and Evolution in Microbial Model Systems, Linnaeus University, Kalmar, Sweden

**Keywords:** diazotroph, growth rates, mortality rates, *in situ* incubations, dilution technique, *nifH*, qPCR

## Abstract

Major advances in understanding the diversity, distribution, and activity of marine N_2_-fixing microorganisms (diazotrophs) have been made in the past decades, however, large gaps in knowledge remain about the environmental controls on growth and mortality rates. In order to measure diazotroph net growth rates and microzooplankton grazing rates on diazotrophs, nutrient perturbation experiments and dilution grazing experiments were conducted using free-floating *in situ* incubation arrays in the vicinity of Station ALOHA in March 2016. Net growth rates for targeted diazotroph taxa as well as *Prochlorococcus, Synechococcus* and photosynthetic picoeukaryotes were determined under high (H) and low (L) nitrate:phosphate (NP) ratio conditions at four depths in the photic zone (25, 45, 75, and 100 m) using quantitative PCR and flow cytometry. Changes in the prokaryote community composition in response to HNP and LNP treatments were characterized using 16S rRNA variable region tag sequencing. Microzooplankton grazing rates on diazotrophs were measured using a modified dilution technique at two depths in the photic zone (15 and 125 m). Net growth rates for most of the targeted diazotrophs after 48 h were not stimulated as expected by LNP conditions, rather enhanced growth rates were often measured in HNP treatments. Interestingly, net growth rates of the uncultivated prymnesiophyte symbiont UCYN-A1 were stimulated in HNP treatments at 75 and 100 m, suggesting that N used for growth was acquired through continuing to fix N_2_ in the presence of nitrate. Net growth rates for UCYN-A1, UCYN-C, *Crocosphaera* sp. (UCYN-B) and the diatom symbiont *Richelia* (associated with *Rhizosolenia*) were uniformly high at 45 m (up to 1.6 ± 0.5 d^-1^), implying that all were growing optimally at the onset of the experiment at that depth. Differences in microzooplankton grazing rates on UCYN-A1 and UCYN-C in 15 m waters indicate that the grazer assemblage preyed preferentially on UCYN-A1. Deeper in the water column (125 m), both diazotrophs were grazed at substantial rates, suggesting grazing pressure may increase with depth in the photic zone. Constraining *in situ* diazotroph growth and mortality rates are important steps for improving parameterization for diazotrophs in global ecosystem models.

## Introduction

Our knowledge of the patterns of marine microbial biodiversity and functional activities has rapidly expanded (Sunagawa et al., 2015; Carradec et al., 2018) with the application of ‘omic’-based techniques. Spatial patterns, however, are ultimately a function of growth and mortality rates of individual taxa, which are processes that are difficult to measure in natural populations. This is especially true for microorganisms responsible for biological nitrogen (N_2_) fixation (diazotrophs) in the marine environment because, despite their importance, they are present at low abundances and many lineages do not have cultivated representatives. Thus, it is not well understood how shifts in the availability of critical nutrients drive changes in the growth (and mortality) of natural populations of diazotrophs.

Marine diazotrophs are diverse, comprised of both phototrophic and heterotrophic Bacteria and Archaea. They vary greatly in morphology and physiology and are expected to have different optimal growth conditions and mortality factors. The filamentous colonial diazotroph *Trichodesmium* and the heterocyst-forming symbionts of diatoms (diatom-diazotroph associations, or DDAs) have long been recognized as important N_2_-fixers. These groups are distributed globally through tropical and subtropical regions, and are a major source of new N in temperate, oligotrophic regions (Villareal, 1992; Capone et al., 2005). Multiple lineages of unicellular N_2_-fixing cyanobacteria are also globally distributed (Moisander et al., 2010; Luo et al., 2012) and are a quantitatively important source of new N (Montoya et al., 2004). This is especially true in the oligotrophic North Pacific subtropical gyre (NPSG) (Dore et al., 2002; Church et al., 2009).

The free-living cyanobacterial diazotroph *Crocosphaera* (UCYN-B) occasionally “blooms” in the NPSG, but arguably the more important unicellular diazotroph in this region is the uncultivated cyanobacteria group A (UCYN-A), which are present year-round in the NPSG. They reach peak abundances in the spring-early summer months and are comprised of diverse sublineages (Thompson et al., 2014; Farnelid et al., 2016; Turk-Kubo et al., 2017). Two of these sublineages, UCYN-A1 and UCYN-A2 are known to live in symbiosis with a prymnesiophyte algae (Thompson et al., 2012; Thompson et al., 2014). Both UCYN-A1 and UCYN-A2 have greatly reduced genomes, having lost important cyanobacterial metabolic capabilities including oxygenic photosynthesis and carbon fixation (Tripp et al., 2010; Bombar et al., 2014). These symbiotic associations are capable of high cellular rates of N_2_ fixation (Martinez-Perez et al., 2016), presumably supported by the transfer of carbon from the host in exchange for reduced N from UCYN-A (Thompson et al., 2012).

Other unicellular cyanobacteria that have been reported include the unicellular cyanobacterial group C (UCYN-C; Foster et al., 2007), which includes some cultivated isolates (Taniuchi et al., 2012), however, little is known about their distribution or importance in the NPSG. *Trichodesmium* and several DDA lineages are also found in the NPSG (Letelier and Karl, 1996; Church et al., 2005a,b, 2009; Sohm et al., 2011b). *Richelia* spp. associated with *Rhizosolenia* (Het-1) and *Hemiaulus* (Het-2), are found in the NPSG and the latter has been identified as the diazotroph that contributes to vertical export following summer blooms (Karl et al., 2012). Diverse non-cyanobacterial diazotrophs have been reported in the NPSG (Bombar et al., 2013; Gradoville et al., 2017), but their distributions and activities, as well as their quantitative significance to biological N_2_ fixation, are not well understood (Turk-Kubo et al., 2014; Bombar et al., 2016; Moisander et al., 2017).

Controls on the N_2_ fixation activity and distribution patters of diazotrophs involve the interplay between the availability of nutrients needed for growth and activity and their ability to compete for these (often limiting) nutrients. Diazotrophic phytoplankton have a competitive advantage over non-diazotrophic phytoplankton when N is limiting, due to their ability to access gaseous N_2_. However, there are various costs associated with fixing N_2_, including a high cellular demand for Fe and the energy required to protect against inhibition of the oxygen-sensitive nitrogenase enzyme in aerobic environments, which are thought to result in a competitive disadvantage over non-diazotrophic phytoplankton when N is available. Under non-Fe limited conditions, diazotrophic phytoplankton are thought to be outcompeted by non-diazotrophic phytoplankton for the limited P at high N:P ratios (Schade et al., 2005). Thus the known biogeography of diazotrophs, at the basin scale, can be best predicted by models that consider the supply ratios of Fe:N and P:N (Ward et al., 2013).

It has been widely assumed that marine N_2_ fixation is inhibited by the presence of nitrate, however, recent discoveries of diazotrophs and measurements of N_2_ fixation rates in nitrate-replete marine environments such as coastal systems (Mulholland et al., 2012; Thompson et al., 2014), regions with seasonal upwelling (Sohm et al., 2011a; Moreira-Coello et al., 2017), mesopelagic waters (Hamersley et al., 2011; Benavides et al., 2018), the Bering (Shiozaki et al., 2017) and Chukchi Seas (Shiozaki et al., 2018) in the Arctic, the Great Belt (Bentzon-Tilia et al., 2015) and oxygen minimum zones (Fernandez et al., 2011), suggests that the influence of DIN availability on diazotroph biogeography and N_2_ fixation is not well-understood. The assumption that DIN inhibits N_2_ fixation is based on several lines of evidence including the competitive disadvantage faced by diazotrophs due to the energetic tradeoff between N_2_ fixation verses nitrate assimilation (Falkowski, 1997), early observations of *Trichodesmium* only in N-depleted surface waters of the oligotrophic ocean (Capone et al., 1997), and direct measurements of nitrate and/or ammonia inhibition of N_2_ fixation in culture experiments (e.g., Mulholland and Capone, 1999; Holl and Montoya, 2005). However, there is increasing evidence that the presence of DIN alone may not exclude diazotroph activity and N_2_ fixation. In culture, evidence is emerging that short-term exposure to nitrate may not inhibit growth or N_2_ fixation in the unicellular diazotroph *Crocosphaera* (Dekaezemacker and Bonnet, 2011) and that N_2_ fixation in *Trichodesmium* is not inhibited at nitrate concentrations typically found in surface waters (Knapp, 2012). Furthermore, the ratio of available N to P may play a critical role, as reduced cellular N_2_ fixation rates at high nitrate concentrations can be offset by increased abundances when P is available in *Trichodesmium* (Knapp et al., 2012). Additionally, continuing to invest in N_2_ fixation in the presence of DIN has been hypothesized as a unconventional strategy to balance redox states (Zehr and Bombar, 2015; Bombar et al., 2016 and references therein).

Cyanobacterial diazotroph net growth rates are a balance between taxa-specific requirements for growth (temperature, light, nutrients), and the ability to compete for limiting nutrients and avoid mortality (grazing and viral lysis). Each of these terms is challenging to evaluate in natural populations, thus predictive models rely heavily on culture-derived information, which is heavily biased toward *Trichodesmium*, to parameterize the N_2_-fixing functional group (Dutkiewicz et al., 2009). Few direct measurements of diazotroph net growth rates have been made in natural populations. Net growth rates of UCYN-A1, *Crocosphaera*, and the gammaproteobacteria γ-24774A11 have been reported to be stimulated by P, Fe+P, and to a lesser extent carbon additions in the South Pacific Ocean (Moisander et al., 2011). However, increases in diazotroph abundances (growth) or the transcription of the *nifH* gene (a proxy for N_2_-fixing activity) are not always observed upon addition of P (Zehr et al., 2007; Turk-Kubo et al., 2012; Krupke et al., 2015; Barcelose Ramos et al., 2017). Turk-Kubo et al. (2015) observed fluctuations in net growth in mesocosms sampled for 23 days, underscoring that this is a dynamic process in which the top-down controls such as grazing and viral lysis are likely to be important factors.

Very little is known about diazotroph mortality in the marine environment. Grazing and viral lysis are assumed to be important factors affecting net growth rates and standing stocks, yet both are difficult to quantify. There have been a number of studies documenting the grazing of different copepod species on *Trichodesmium* (O’Neil et al., 1996; O’Neil, 1998), UCYN-A (Scavotto et al., 2015; Conroy et al., 2017), UCYN-B (Conroy et al., 2017), UCYN-C (Hunt et al., 2016) and *Richelia* associated with both *Rhizosolenia* (Het-1) and *Hemiaulus* (Het-2) (Hunt et al., 2016; Conroy et al., 2017), through direct observation or detection in full-gut copepods. Collectively, these studies indicate that direct transfer of diazotroph derived N to mesozooplankton (>200 μm) is potentially an important factor in marine food webs.

There are few direct observations of microzooplankton (<200 μm) grazing on diazotrophs, despite their prominent role as primary consumers in the oligotrophic open ocean (Schmoker et al., 2013). A recent study measured microzooplankton grazing rates on *Crocosphaera* during a summertime *Crocosphaera* bloom in the NPSG and concluded that grazing and viral lysis were likely controlling their abundances, rather than bottom-up factors (Wilson et al., 2017). Wilson et al. (2017) used a modification of the dilution technique developed by Landry and Hassett (1982) and flow cytometry to measure growth rates. This technique has been widely used to measure microzooplankton grazing rates on pico- and nanoplankton communities (0.2–2 μm and 2–20 μm size classes, respectively) in the ocean, and the underlying assumptions and limitations have been discussed in detail elsewhere (Landry et al., 1995; Schmoker et al., 2013). Growth and mortality rates of the entire phytoplankton community can be calculated using changes in chl *a* during the incubation, however, this approach can also be used to measure these parameters on individual taxa such as *Prochlorococcus* and *Synechococcus* (Chen et al., 2009), as well as *Crocosphaera* (Wilson et al., 2017).

In order to measure how shifts in the available nutrients influence the growth of natural diazotroph populations, free-floating *in situ* incubation arrays, originally described in Böttjer et al. (2017), were used to investigate net growth rates of phototrophic picoplankton and diazotrophic taxa as a function of depth and N:P ratio. In addition, microzooplankton grazing rates on diazotrophs were measured using a modified dilution method (Landry et al., 1995), at two depths in the photic zone (15 and 125 m) using a combination of *in situ* floating arrays and deckboard incubations. Diazotroph net growth rates were often stimulated by HNP conditions, which was an unexpected result, and the depth at which HNP conditions were favorable was taxa-dependent. Microzooplankton grazing pressure on the two most abundant diazotrophs, UCYN-A1 and UCYN-C, increased with depth, which was consistent with the pattern observed for the total phytoplankton community.

## Materials and Methods

### Experimental Incubations

Five experiments were conducted as part of this study. Changes in diazotroph growth rates in response to shifts in available nutrient resource ratios were measured in growth experiment 1 (G1). Two experiments, grazing dilution experiment 1 (GR1) and 2 (GR2), were conducted to measure microzooplankton grazing rates on specific diazotrophic taxa, using a modified dilution technique amended with N, P and Fe (Landry et al., 1995). In parallel to the GR1 and GR2 experiments, smaller scale dilution experiments (GR1-N and GR2-N) amended only with P and Fe were conducted, based on the assumption that the addition of N substrates may inhibit diazotroph growth.

All experiments were conducted in the core of an anticyclonic eddy situated to the east of Station ALOHA in the NPSG between March 24 and 28, 2016 from aboard the R/V Kilo Moana (cruise no. KM1605). Seawater from the appropriate depths was collected for all experiments using a rosette of Niskin bottles coupled to a SeaBird CTD detector. Handling of water for each experiment type differed, and is detailed in sections below, however, all experiments were conducted in small (2.3 or 4 L) bottles, which have been recently shown to bias against large (20–200 μm) diazotrophic taxa (Follett et al., 2018). Experiments G1 and GR2 were incubated at fixed depths as part of a free-drifting array approach based on Böttjer et al. (2017), that makes it possible to incubate at *in situ* temperatures and irradiances. GR1, GR1-N, and GR2-N were incubated in simulated *in situ* light and temperature conditions in an on-deck incubator. Dates, experimental details and environmental parameters specific to each experiment are detailed in **Table 1**.

**Table 1:** Growth (G1) and grazing rate (GR1, GR2) experiment details.

	GR1	GR1-N	G1	GR2	GR2-N
Date	March 24–25, 2016	March 24–25, 2016	March 25–27, 2016	March 27–28, 2016	March 27–28, 2016
Lat	22°23.799′ N	22°23.799′ N	22°23.997′ N	22°33.276′ N	22°33.276′ N
Long	156°35.817′ W	156°35.817′ W	156°35.741′ W	156°33.190′ W	156°33.190′ W
Experiment type	Grazing (5 pt. dilution)	Grazing (-N; 3 pt. dilution)	Growth rates (HNP vs. LNP)	Grazing (4 pt. dilution)	Grazing (-N; 2 pt. dilution)
% dilution levels	20,40,60,80,100	20,60,100	*na*	20,40,80,100	60,100
Incubation method	Deckboard	Deckboard	*In situ* array	*In situ* array	Deckboard
Depth(s) (m)	15	15	25, 45, 75, 100	15, 125	15
Incubation period (h)	24	24	48	24	24
MLD (m)	53 m	53 m	87 m	65 m	65 m
DCM (m)	140 m	140 m	140 m	140 m	140 m
Nitrocline (m)	125 m	125 m	125 m	125 m	125 m


*MLD, mix layer depth; DCM, deep chlorophyll maximum; na, not applicable.*

#### Diazotroph Net Growth Rate Incubations (G1)

Seawater collected from 25, 45, 75, and 100 m was dispensed directly from the Niskin bottles into 4 L polycarbonate bottles, taking measures to randomize the filling of the bottles. The polycarbonate bottles were acid-washed with trace metal clean HCl prior to each experiment, however, these incubations are not considered trace metal clean. No pre-filtration to remove grazers was used, so measured growth rates determined from changes in cell abundances over the length of the incubation incorporate cell death, and are therefore net growth rates.

Replicate bottles from each depth were amended with either NaNO_3_ (high N:P, HNP) or KH_2_PO_4_ (low N:P; LNP) for final concentrations of 2 and 0.5 μM, respectively. All incubation bottles, including the controls (C), received FeCl_3_ at a final concentration of 10 nM. Fe was added to ensure that the response of the diazotrophic community was to changes in the N:P ratio under non-Fe limiting conditions, as Fe is generally not considered to limit primary production at Station ALOHA (Boyle et al., 2005). Water was collected around 2 a.m. and the 4 L bottles were kept in the dark until the array was deployed before dawn. T_0_ samples were immediately subsampled for phototrophic picoplankton cell counts using flow cytometry, nutrients, chl *a* and DNA. The G1 array was incubated *in situ* for 48 h, with bottles floating at the depths from which the water was originally sampled. The array deployment and recovery occurred at dawn, to minimize manipulating the communities in daylight. All bottles were kept in the dark until they were subsampled.

#### Incubations to Measure Microzooplankton Grazing Rates on Diazotrophs (GR1 and GR2)

For the dilution experiments GR1 and GR2, seawater from the appropriate depth was transferred into acid-washed 23 L polycarbonate carboys, which were then gently combined into a 50 L carboy to ensure homogeneity between treatments. Filtered seawater (FSW) for diluent was prepared by filtering experimental seawater through a Pall 0.2 μm Acropak 1550 Capsule Filter with Supor Membrane (Pall Corp, Port Washington, NY, United States), and was stored at room temperature in the dark while the experiment was setup. FSW was combined with whole sea water (WSW) in 2.3 L incubation bottles at ratios described below for each experiment and in **Table 1**. The bottles were amended with N, P and Fe at final concentrations of 2.0 μM NO_3_^-^, 0.2 μM NH_4_^+^, 0.5 μM PO_4_^3-^, and 0.1 μM Fe. For each experiment, a control set of triplicate bottles containing 100% WSW were not nutrient-amended in order to determine net growth rates during the incubation period. T_0_ samples were taken from the 50 L carboy and immediately subsampled for chl *a* and DNA. All dilution experiments were initiated at night, to minimize photoadaption or light shock.

GR1 consisted of five dilution levels, 20, 40, 60, 80, and 100% WSW. In parallel with GR1, a smaller-scale dilution experiment (GR1-N) was conducted that consisted of three dilution levels (20, 60, and 100% WSW) in triplicate 1.2 L bottles. Triplicate 1.2 L bottles containing 100% WSW were not amended with nutrients as noted above. The remaining bottles were amended with only P and Fe at final concentrations of 0.5 μM PO_4_^3-^, and 0.1 μM Fe. All bottles (GR1 and GR1-N) were incubated for 24 h in simulated *in situ* light and temperature conditions in an on-deck incubator with a continuous flow of surface seawater to simulate conditions at 15 m. At T_24_, all bottles were subsampled for chl *a* and DNA.

GR2 consisted of four dilution levels at each depth, 20, 40, 80, and 100% WSW. Incubation bottles were deployed on the *in situ* incubation array at 15 and 125 m and incubated for 24 h. As with GR1, a smaller-scale dilution experiment (GR2–N) was conducted in parallel with GR2, but only with 15 m water. GR2-N consisted of two dilution levels (60 and 100% WSW) in triplicate 1.2 L bottles. Nutrient amendments and WSW control bottles were consistent with GR2-N described above. The GR2-N bottles were incubated as described above for GR1-N. At T_24_, all bottles were subsampled for chl *a* and DNA.

### Quantifying Diazotroph Abundances Using Quantitative PCR and Calculating Net Growth and Mortality Rates

Samples for DNA extraction were gently filtered using peristaltic pumps through Sterivex filters (Millipore) for growth rate experiments or 0.2 μm pore-size 25 mm diameter Supor filters (Pall Corp, Port Washington, NY, United States) in sterile swinnex holders (Millipore) for grazing experiments. Sterivex filters were immediately sealed, and Supor filters were transferred into 2.0 mL bead beating tubes filled with 0.1 and 0.5 mm sterile glass beads. Filters were flash frozen in liquid nitrogen and stored at -80°C until extraction. Just prior to DNA extraction, the sterivex cartridges were broken and the filters were transferred into the bead beating tubes described above.

DNA was extracted using a protocol chosen for high quality recovery of DNA from algal cells. Details of this protocol are described in Moisander et al. (2008). Briefly, cells were disrupted using freeze-thaw, bead beating, and proteinase K digestion steps prior to a Qiacube^®^ (Qiagen) automated on-column DNA extraction and clean-up protocol using DNeasy kit (Qiagen) components. Concentration and quality of purified DNA were measured using a NanoDrop (Thermo Scientific, Waltham, MA, United States). Extracted DNA was stored at -20°C until use.

Abundances of nine diazotrophic taxa were determined using quantitative PCR (qPCR) assays based on Taqman^®^ chemistry. Primers and probes targeting the following diazotrophic taxa were used: unicellular cyanobacterial group A1 (UCYN-A1; Church et al., 2005a), unicellular cyanobacterial group A2/A3 (UCYN-A2/A3; Thompson et al., 2014), *Crocosphaera* (UCYN-B: Moisander et al., 2010), *Cyanothece*-like organisms (UCYN-C; Foster et al., 2007), *Trichodesmium* spp. (Church et al., 2005a), *Richelia* associated with *Rhizosolenia* (Het-1; Church et al., 2005b), *Richelia* associated with *Hemiaulus* (Het-2; Foster et al., 2007), *Calothrix* associated with *Chaetoceros* (Foster et al., 2007), and a gammaproteobacterial group (γ-24774A11; Moisander et al., 2008). All nine taxa were quantified in G1, but only the most abundant taxa present in the water at the time of the experimental work (UCYN-A1 and UCYN-C) were quantified in the grazing rate experiments.

Details of qPCR standard generation, plate design, thermocycling parameters, inhibition tests, determination of the limit of detection (LOD) and quantification (LOQ), as well as abundance calculations are described in Goebel et al. (2010). The LOD and LOQ for G1 and GR1 were 13 and 100 *nifH* copies L^-1^, respectively. The LOD and LOQ for GR2 were between 33–50 and 267–400 *nifH* copies L^-1^, respectively.

Net growth rates from G1 were calculated as in Turk-Kubo et al. (2015) using the equation *k* = 2.303^.^[log_10_(N_t2_/N_t1_)]/(t_2_-t_1_) where N_x_ = abundance at time × (t_x_), and assuming that there is a single *nifH* copy per cell. It should be noted that there is evidence of multiple genome copies in natural populations of *Trichodesmium* (Sargent et al., 2016), and little is known about genome copy numbers in natural populations of other diazotroph taxa. Therefore, growth rates based on qPCR may be impacted if the number of genome copies is variable across time in a given population. However, based on the strong linear relationship between cell counts L^-1^ and *nifH* copies L^-1^ across variable sampling times reported by Sargent et al. (2016), the number of genome copies appears to be consistent, at least for *Trichodesmium*.

Net growth rates in G1 were determined for UCYN-A1, UCYN-B, UCYN-C in all depths and treatments, but only in surface waters for Het-1 (25 and 45 m) and Het-2 (25 m), due to low abundances for these latter taxa lower in the water column. UCYN-A2/A3, *Trichodesmium*, Het-3 and γ-24774A11 were often detectable, but abundances were too low for establishing reliable rates (detected, not quantified; DNQs). Comparisons between treatments and the controls were made using one way analysis of variance, followed by testing independent variables with a pairwise *t*-test in R (R Development Core Team, 2012).

Mortality rates (*m*) on individual diazotroph taxa in GR1, GR1-N, GR2, and GR2-N were calculated using the slope of Model I linear regressions of qPCR-based diazotroph apparent growth rates in each nutrient-amended dilution vs. the dilution level, using the established convention that a negative slope indicated positive grazing pressure (Landry and Hassett, 1982). Nutrient enriched growth rates (μ_n_) of each prey were determined using the y-intercept of the regression of nutrient-amended treatments, which represents a theoretical scenario where all grazing pressure is removed from the incubations. Intrinsic (unenriched) growth rates (μ_0_) were calculated from the nutrient-enriched intrinsic growth rates, corrected for differences in growth rates between amended and unamended treatments of 100% WSW (Landry et al., 1995). Mortality rates are reported only when the linear regressions were significant (*p* ≤ 0.05), otherwise they are reported as non-significant (ns).

### Phototrophic Picoplankton Community Composition and Net Growth Rates in G1

Subsamples (2 mL) for the enumeration of *Prochlorococcus, Synechococcus* and photosynthetic picoeukaryotes (PPEs) were collected from G1 at times T_0_ and T_48_ and immediately fixed with electron microscopy grade gluteraldehyde (Electron Microscopy Sciences, Hatfield, PA) for a final gluteraldehyde concentration of 0.25% v/v. Samples were fixed in the dark for 15 min, then flash frozen in liquid nitrogen and stored at -80°C until processing. Cells were enumerated using a BD Influx cell sorter with a 488 nm Sapphire laser (Coherent, Santa Clara, CA, United States). Large particles were removed from samples prior to counting using a CellTrics^®^ filter with 30 μm mesh (Partec, Swedesboro, NJ, United States). *Synechococcus* cells were identified based on their phycoerythrin signal (orange fluorescence), and non-phycoerythrin containing cells (*Prochlorococcus* and PPEs) were identified using chl *a* fluorescence (red florescence) and forward scatter (FSC; a proxy for cell size). Data was triggered on the FSC channel, events were counted for 10 min, and data was processed using FlowJo v10.0.7 (Tree Star, Inc., Ashland, OR, United States). Net growth rates (G1) were calculated from abundances as described above.

### Measuring Shifts in Microbial Community Composition During G1 Using 16S rRNA Gene Tag Sequencing

Bacterial community composition was characterized using the V3/V4 hypervariable region of the 16S-rRNA gene. Universal primers targeting Bacteria, Bakt_341F and Bakt_805R (Herlemann et al., 2011) were modified with common sequence linkers (Moonsamy et al., 2013) to facilitate library preparation and sample barcoding. PCR amplifications were carried out in triplicate reactions for each sample, using reaction conditions and thermocycling parameters detailed in Shilova et al. (2017). Libraries were prepared at the DNA Services Facility at the University of Illinois, Chicago, using the targeted amplicon sequencing approach described in Green et al. (2015). Paired-end reads were sequenced using Illumina MiSeq technology at the W.M. Keck Center for Comparative and Functional Genomics at the University of Illinois at Urbana-Champaign. De-multiplexed raw paired-end reads were merged using PEAR (Zhang et al., 2014). QIIME (Caporaso et al., 2010) was used for quality filtering (phred score of 20), de novo chimera removal, operational taxonomic unit (OTU) determination (97% nucleotide similarity) using the usearch6.1 clustering method (Edgar, 2010), and assigning the taxonomy of representative sequences using the Silva reference database release 104 (Quast et al., 2013) ^1^. Raw reads were deposited in the Sequence Read Archive at National Center for Biotechnology Information^2^ under BioSample Project ID PRJNA453423.

Community composition data was analyzed using the R package Phyloseq (McMurdie and Holmes, 2013). Data was subsampled using the following criteria: (i) removing OTUs not seen more than 100 times; and (ii) transforming to an equal sampling depth of 15,400 sequences; (iii) subsample for the top 20 most abundant OTUs. Ecological distances between samples based on community composition was determined using Jaccard and Bray–Curtis ecological indices on subsampled data and Principal coordinate analysis (PCoA) was performed on the resulting distance matrices, to visualize the dissimilarity between samples and community composition.

### Chlorophyll *a* and Nutrient Sample Collection and Processing

For chl *a* concentrations from G1, between 250 and 500 mL of seawater was gently filtered under vacuum onto GF/F filters (Whatman^®^, Buckinghamshire, United Kingdom) and measured using the non-acidified protocol originally described in Welschmeyer (1994) using a Turner 10-AU fluorometer (San Jose, CA, United States).

Nutrient samples from the water column [nitrate + nitrite and inorganic phosphate (PO_4_^3-^)] were collected and analyzed consistent with the Hawaii Ocean Time (HOT) Series Program^3^ at the University of Hawaii, Manoa.

## Results

### Initial Environmental Conditions and Microbial Community Composition

**FIGURE 1:**
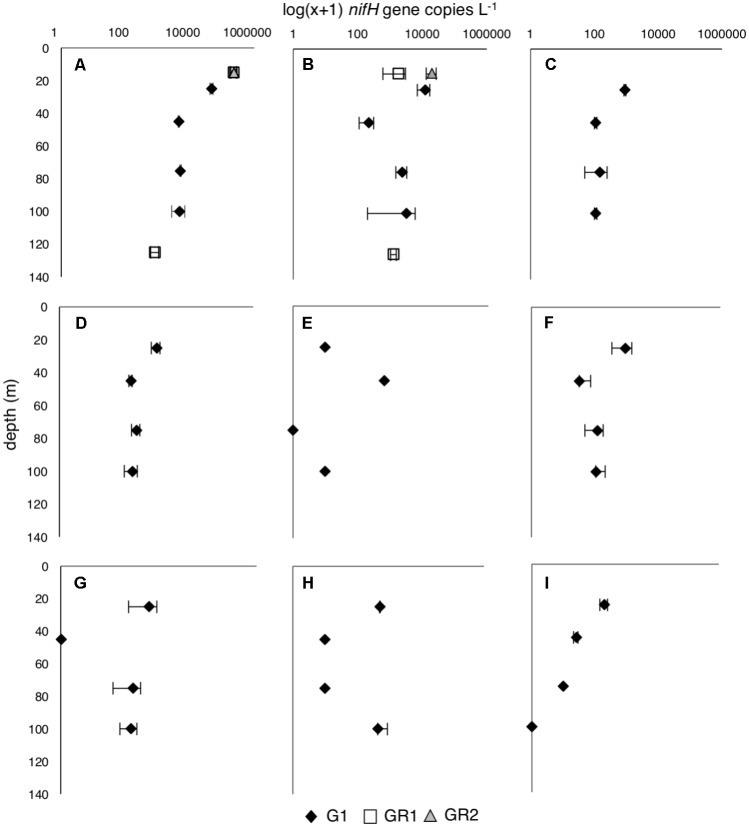
Diazotroph abundances [log(x+1) *nifH* copies L^-1^] throughout the water column at the beginning of growth rate (G1) and dilution grazing rate (GR1 and GR2) experiments. **(A)** UCYN-A1, **(B)** UCYN-C, **(C)** UCYN-A3, **(D)** UCYN-B, **(E)** *Trichodesmium*, **(F)** Het-1; **(G)** Het-2, **(H)** Het-3; **(I)** γ-24774A11. Only the most abundant diazotrophs, UCYN-A1 and UCYN-C, were measured for GR1 and GR2. Error bars are replicate incubations.

All three experiments, G1, GR1, and GR2, were conducted during spring 2016 in the NPSG with water collected at the center of an anticyclonic eddy characterized by enhanced chlorophyll located 60 km north of Molokai (22.5° N and 156.5° W). The eddy appeared to be decreasing in strength at the time of sampling, with low and variable currents at the center. The mixed layer depth and depth of the deep chlorophyll maximum (DCM) were apparent at 87 and 140 m, respectively (**Table 1**). Nitrate + nitrite concentrations were very low in the top 100 m of the water column at 2.8 ± 3.2 nM, while PO_4_^3-^ concentrations were 0.11 ± 0.01 μM. *Prochlorococcus* abundances at G1 T_0_ were 7.2 × 10^4^ ± 6.9 × 10^3^ cells mL^-1^ at 25 m and increased slightly to 9.6 × 10^4^ ± 5.2 × 10^2^ cells mL^-1^ at 100 m; *Synechococcus* and photosynthetic picoeukaryote (PPE) abundances were consistent throughout the top 100 m at 7.5 × 10^2^ ± 4.8 × 10^1^ cells mL^-1^ and 1.5 × 10^3^ ± 4.7 × 10^2^ cells mL^-1^, respectively. The most abundant diazotrophs were unicellular cyanobacterial phylotypes UCYN-A1 and UCYN-C, which had maximum abundances of 4.7 × 10^4^ and 1.2 × 10^4^ *nifH* copies L^-1^, respectively. All other targeted diazotrophs were detected in the water column, but at much lower abundances than UCYN-A1 and UCYN-C (**Figure 1**), including the large (20–200 μm) diazotrophic taxa (*Trichodesmium* and DDAs), which is consistent with known annual patterns in their abundances (Church et al., 2009). However, it cannot be ruled out that these abundances are underestimated given a recent report that small bottle incubations may bias against the larger diazotrophs, presumably due to heterogeneity in these populations resulting from buoyancy and vertical migration (Follett et al., 2018).

### Net Growth Rates in Photosynthetic Microbial Populations Exhibited Taxa- and Depth-Specific Responses to Different Nutrient Ratios

**FIGURE 2:**
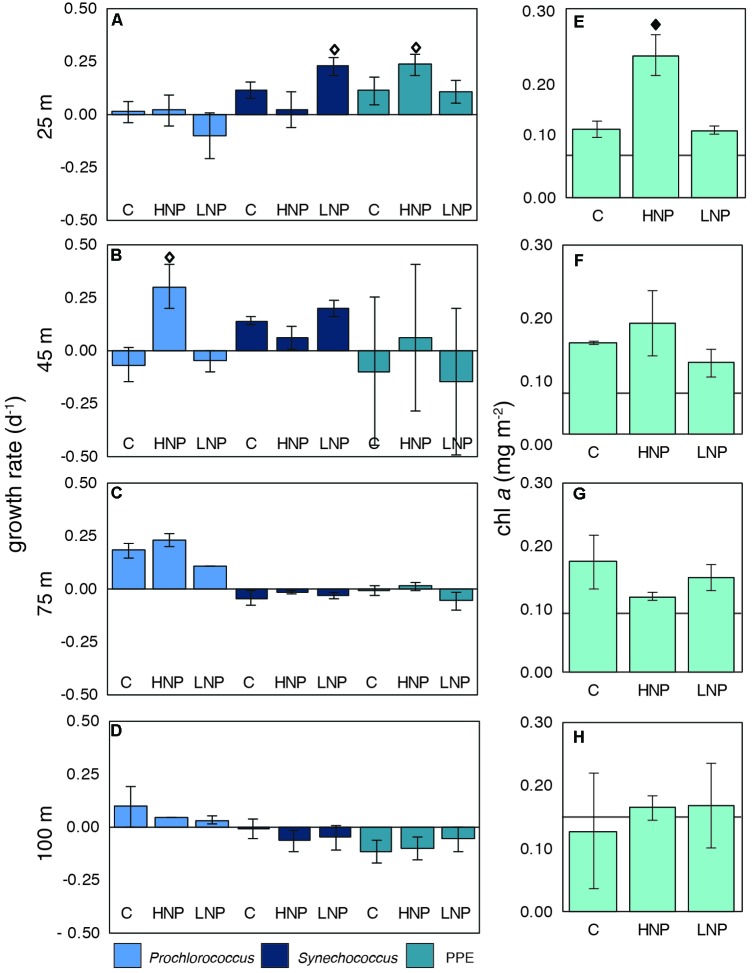
Net growth rates of photosynthetic microbial populations **(A–D)** and biomass changes (chl *a*; **E–H**) at four depths [25 **(A,E)**, 45 **(B,F)**, 75 **(C,G)** and 100 m **(D,H)**] in the photic zone during experiment G1. chl *a* concentrations at T_0_ are indicated with a line. Error bars are replicate standard deviations. Statistically significant differences between control and treatment are indicated with a solid diamond (*p* ≤ 0.01) or an open diamond (*p* ≤ 0.05). C, control; HNP, high N:P treatment; LNP, low N:P treatment; PPE, photosynthetic picoeukaryote.

The responses of the major photosynthetic populations, *Prochlorococcus, Synechococcus*, and PPEs to HNP and LNP conditions during G1 were characterized using net growth rates derived from FCM-based cell counts (**Figures 2A–D**) as well as changes in chl *a* concentrations (as a proxy for photosynthetic biomass; **Figures 2E–H**). Over the course of the experiment, chl *a* content increased in all treatments and at all depths except at 100 m, where T_0_ and T_48_ chl *a* concentrations were similar (**Figures 2E–H**). Significant (*p* ≤ 0.01) increases in chl *a* content with respect to control concentrations were measured only in the HNP treatment at 25 m (an approximately 2-fold increase; **Figure 2E**).

Increases in chl *a* concentrations in the 25 m HNP treatment may be due to a significant (*p* ≤ 0.05) stimulation of biomass from the PPE population compared to T_48_ controls (**Figure 2A**). This was the only depth and treatment where the PPE population had higher net growth rates than the controls, indicating that the PPE population was N-limited in surface waters. *Synechococcus* growth rates were highest in the LNP treatment at 25 m, implying that these populations were P-limited at the initiation of the experiment, despite ambient PO_4_^3-^ concentration of 0.11 ± 0.01 μM in the top 100 m of the water column (**Figure 2A**). In contrast, *Prochlorococcus* growth rates were significantly (*p* ≤ 0.05) stimulated only in HNP treatments at 45 m (**Figure 2B**).

### Diazotroph Net Growth Rates Exhibit Taxa- and Depth-Specific Responses to Different Nutrient Ratios (G1)

**FIGURE 3:**
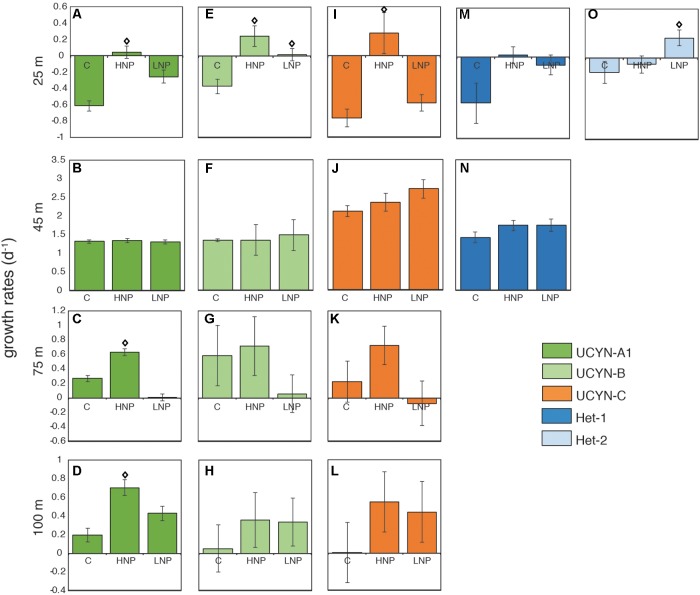
Diazotroph net growth rates throughout the photic zone during experiment G1 at 25 m **(A,E,I,M,O)**, 45 m **(B,F,J,N)**, 75 m **(C,G,K)**, and 100 m **(D,H,L)**. Error bars are replicate standard deviations. Statistically significant differences between control and treatment are indicated with an open diamond (*p* ≤ 0.05). C, control; HNP, high N:P treatments; LNP, low N:P treatment.

Despite relatively stable abundances throughout the top 100 m (**Figure 1**), individual diazotrophic taxa exhibited depth-dependent responses to changes in nutrient ratio (**Figure 3** and **Supplementary Table S1**). In surface waters (25 m), all of the diazotrophs had negative growth rates in the control treatment (**Figures 3A,E,I,M,O**). In 25 m HNP treatments, UCYN-B and UCYN-C had positive and enhanced growth rates (with respect to the control treatment) of 0.25 ± 0.07 d^-1^ (**Figure 3E**) and 0.27 ± 0.10 d^-1^ (**Figure 3I**), respectively; other groups had negative or close to zero growth rates. The only diazotroph that had enhanced growth rates in the LNP treatment at this depth was Het-2 at 0.24 ± 0.10 d^-1^ (**Figure 3O**).

Surprisingly, the most abundant diazotrophs, UCYN-A1, UCYN-B, UCYN-C and Het-1 had high net growth rates across all controls and treatments at 45 m (**Figures 3B,F,J,N**), despite all having peak abundances at 25 m (**Figure 1**). UCYN-A1 and UCYN-B growth rates were uniformly high and insensitive to nutrient additions (1.32 ± 0.02 d^-1^ and 1.39 ± 0.08 d^-1,^ respectively; **Figures 3B,F**), suggesting that these taxa were growing optimally at this depth at the start of the experiment, and growth was not inhibited or enhanced by experimental treatments. In contrast, both UCYN-C and Het-1 growth rates were slightly enhanced in LNP treatments (**Figures 3J,N**). UCYN-C growth rates in the control (2.11 ± 0.24 d^-1^) were less than those measured in LNP treatments (2.71 ± 0.26 d^-1^), and the same was true for Het-1 (control 1.42 ± 0.17 d^-1^ vs. LNP 1.74 ± 0.11 d^-1^). These results suggest UCYN-C and Het-1 have a competitive advantage in the N-limited conditions at this depth.

Deeper in the water column (75 and 100 m incubations), net growth rates could only be determined for the unicellular taxa. There was no significant difference in net growth rates between control and LNP/HNP treatments at these depths for UCYN-B and UCYN-C (**Figures 3G,H,K,L**). However, UCYN-A1 net growth rates were significantly stimulated (*p* ≤ 0.05), with respect to control incubations, under HNP conditions at 75 m (0.63 ± 0.05 d^-1^; **Figure 3C**) and 100 m (0.70 ± 0.08 d^-1^; **Figure 3D**).

### Shifts in Prokaryote Microbial Community Composition During G1

**FIGURE 4:**
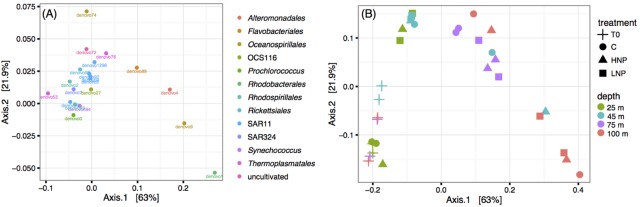
Principal coordinate analysis using the Jaccard ecological index to determine dissimilarity between samples based on microbial community composition. Data was transformed to equal sampling depth and OTUs were defined at 97% nucleotide similarity. The 20 most abundant OTUs **(A)** and community composition of each experimental bottle **(B)**. C, control; HNP, high N:P treatments; LNP, low N:P treatment.

16S rRNA gene variable region amplicon tag sequencing was used to determine whether there were significant changes in microbial community composition. *Prochlorococcus* (21.8–51.3% of sequences per sample) and SAR11 (10.0–16.6% of sequences per sample) dominated the prokaryote community throughout the experiment (**Supplementary Table S2**). T_0_ samples showed that there was a relatively constant microbial community within the top 100 m (**Figure 4A**), consistent with the presence of a deep mixed layer at the time of the experiment. Furthermore, there were no large shifts in relative abundances of these taxa in response to HNP and LNP treatments, including *Prochlorococcus* at 45 m, indicating that although cell numbers increased, the relative proportion of *Prochlorococcus* to other microbes (mainly SAR11) remained stable. Even at the finest taxonomic resolution, *Prochlorococcus* oligotypes (Eren et al., 2013) were also consistent across depths and treatments (data not shown).

There were minor changes in community composition between T_0_ and T_48_ at 75 and 100 m depths, but not between experimental treatments (**Figure 4B**). Ordination analysis using the Jaccard ecological index indicated that community shifts were driven by increases in relative abundances of the following four taxa in all treatments: *Rhodobacterales* (denovo1), *Alteromonadales* (denovo4), *Oceanospirillales* (denovo9), and *Flavobacteriales* (denovo89) (**Figure 4A**). These taxa are frequently found in associations and on particles and are known to respond quickly to incubation conditions (Fontanez et al., 2015). It is important to note that treatments often did not cluster together, indicating that changes in nutrient ratios did not drive any consistent patterns; T_48_ samples clustered according to depth (**Figure 4B**). Furthermore, there was not a correlation between relative abundances and diazotroph net growth rates in samples where a diazotroph had differential responses to HNP/LNP treatments, e.g., UCYN-A1 at 75 or 100 m.

### Microzooplankton Grazing Pressure on UCYN-A1 and UCYN-C Was Highest Near the Deep Chlorophyll Maximum

**FIGURE 5:**
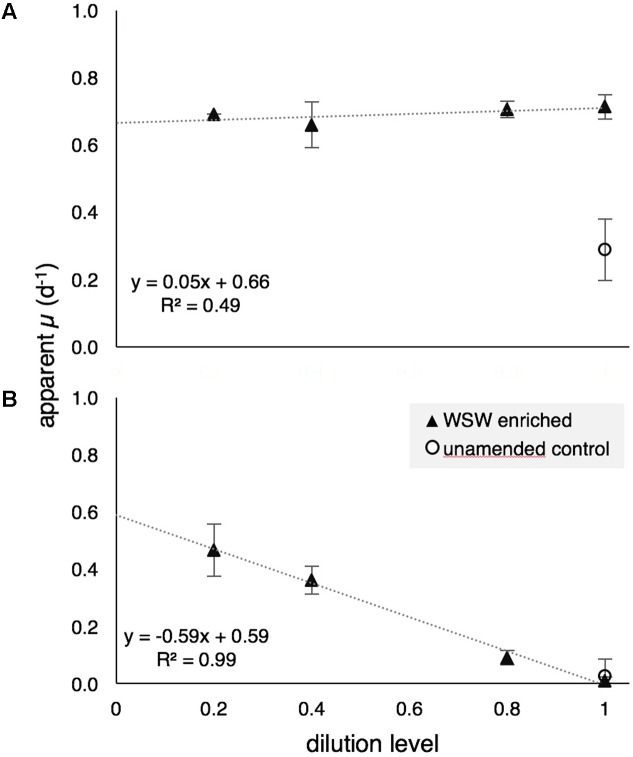
Apparent growth rates (μ) of the bulk photosynthetic community (based on chl *a*) at 15 m **(A)** and 125 m **(B)** during modified dilution rate experiments GR2. Positive grazing mortality (*m)* at 125 m is evidenced by the negative slope of the model I linear regression. WSW, whole sea water.

During GR2, grazing rates on the bulk phytoplankton community, based on changes in chl *a*, indicated that there was no detectable grazing pressure in surface waters (**Figure 5A**), while grazing pressure was significant near the DCM (125 m; **Figure 5B**). The measured mortality rate (*m*) on the bulk phytoplankton community at 125 m was 0.60 d^-1^, which is higher than the mean rate for this region (0.29 d^-1^) reported in Schmoker et al. (2013). Enriched (μ_n_) and intrinsic (μ_0_) growth rates at 15 m were 0.66 and 0.24 d^-1^, respectively, indicating that the addition of nutrients stimulated phytoplankton growth rates in surface waters. The community seemed less responsive to added nutrients at 125 m, with μ_n_ and μ_0_ of 0.59 and 0.62 d^-1^, respectively.

**FIGURE 6:**
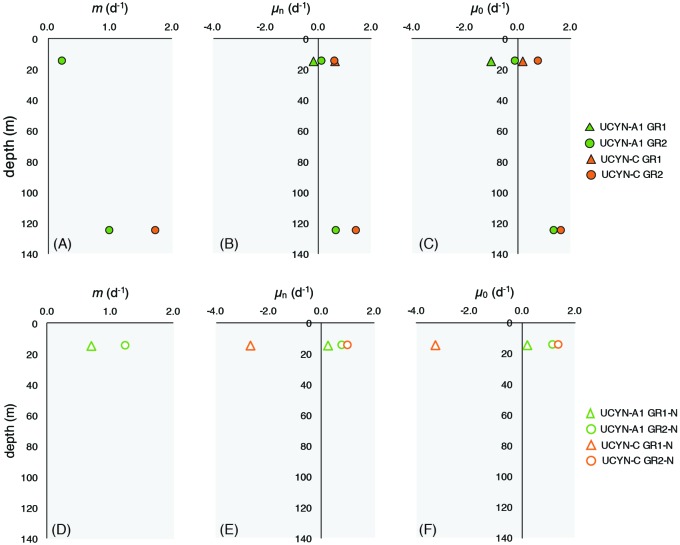
Microzooplankton grazing rates on UCYN-A1 and UCYN-C during modified dilution grazing rate incubations GR1 and GR2 **(A–C)** and GR1-N and GR2-N **(D–F)**. Mortality rates (*m*) are reported only when *p* ≤ 0.05. UCYN-A1 mortality rates in GR1 were non-significant. UCYN-C mortality rates were non-significant at 15 m for all experiments *m*, grazing mortality rate; μ_n_, enriched growth rate; μ_0_, intrinsic growth rate.

Patterns of grazing mortality specifically for UCYN-A1 and UCYN-C were consistent with the depth-dependent patterns observed for the phytoplankton community. In GR1, no significant grazing pressure was measured at 15 m for either diazotroph taxa, however, variable mortality rates on UCYN-A1, and UCYN-C were measured during GR2 (**Figure 6**). In GR2, grazing rates on UCYN-A1 were lower at the surface (15 m; 0.24 d^-1^) than deeper in the photic zone (125 m; 1.00 d^-1^; **Figure 6**). UCYN-C did not seem to have significant grazing pressure at 15 m, while at 125 m, mortality rates were high (1.75 d^-1^). Enrichment with nutrients did appear to stimulate UCYN-A1 growth rates at 15 m, with enriched (μ_n_) and intrinsic (μ_0_) growth rates of 0.16 and -0.13 d^-1^, respectively. However, this was not the case for samples collected deeper in the water column for either taxa. Grazing rates for other diazotrophs were not measured due to their low abundances in T_0_ samples. Together, the chl *a*-based phytoplankton mortality rates and qPCR-based diazotroph mortality rates indicated that grazing pressure was less intense near the surface than deeper in the water column.

Grazing dilution incubations with water from 15 m were conducted during GR1 and GR2, using a nutrient addition that did not include additional fixed N (GR1-N and GR2-N), as the presence of N has been shown to inhibit diazotroph growth in cultures (e.g., Holl and Montoya, 2005). Mortality rates on UCYN-A1 in GR2–N and GR1–N were higher than in the typical modified dilution rate experiments at 1.25 and 0.69 d^-1^, respectively (**Figure 6**). In these experiments, enriched growth rates for UCYN-A1 were positive with μ_n_ ranging between 0.28 and 0.82 d^-1^. No significant mortality rates were measured for UCYN-C in these experiments. These findings are inconsistent with growth rates measured in G1 where growth rates at 25 m were negative in response to P additions (LNP treatment).

## Discussion

### Diazotrophs Are Capable of High *in Situ* Growth Rates

Early culture-based studies suggested that diazotrophs should have slower growth rates than their non-diazotrophic counterparts in natural populations (Fu and Bell, 2003; Falcon et al., 2004; LaRoche and Breitbarth, 2005). However, this assumption was challenged when Moisander et al. (2011) reported net growth rates from field populations of *Crocosphaera* in the South Pacific, which were higher than culture-based growth rate measurements (**Table 2**). *In situ* net growth rates were also unexpectedly high during a perturbation mesocosm study in the southwestern lagoon of New Caledonia (Turk-Kubo et al., 2015), where maximum net growth rates for unicellular and heterocyst-forming diazotrophs were as high as 2.2 d^-1^ (**Table 2**).

**Table 2:** Compilation of diazotroph growth rates measured *in situ*, along with selected culture-based measurement.

Diazotroph	Maximum net growth rate d^-1^	Method	Location	Experimental Conditions or *in situ*	Reference
UCYN-A^a^	0.4	^13^C	Station ALOHA	*In situ*	Thompson et al., 2012
UCYN-A^a^	0.5	^13^C	North Atlantic	*In situ*	Krupke et al., 2015
UCYN-A^a^	0.8	cell size, modeled	North Pacific	*In situ*	Goebel et al., 2008
UCYN-A (small)^a^	0.6^c^	^13^C	North Atlantic	*In situ*	Martinez-Perez et al., 2016
UCYN-A (large)^b^	1.1^c^	^13^C	North Atlantic	*In situ*	Martinez-Perez et al., 2016
UCYN-A1	1.3	qPCR	NPSG	HNP treatment; 45 m	*This study*
UCYN-A1	1.3	qPCR	NPSG	LNP treatment; 45 m	*This study*
UCYN-A1	1.3	qPCR	NPSG	C treatment; 45 m	*This study*
UCYN-A1	0.2	qPCR	South Pacific	Fe and P-Fe treatments	Moisander et al., 2011
UCYN-A1	0.7	qPCR	Southwest Pacific	P treatment	Turk-Kubo et al., 2015
UCYN-A2	1.7	qPCR	Southwest Pacific	P treatment	Turk-Kubo et al., 2015
UCYN-B	1.5	qPCR	NPSG	LNP treatment; 45 m	*This study*
UCYN-B	1.3	qPCR	NPSG	HNP treatment; 45 m	*This study*
UCYN-B	1.3	qPCR	NPSG	C treatment; 45 m	*This study*
UCYN-B	0.6	qPCR	South Pacific	Fe and P-Fe treatments	Moisander et al., 2011
UCYN-B	1.4	qPCR	Southwest Pacific	P treatment	Turk-Kubo et al., 2015
UCYN-B	0.6	FCM	NPSG	*In situ*	Wilson et al., 2017
UCYN-C	2.7	qPCR	NPSG	LNP treatment; 45 m	*This study*
UCYN-C	2.4	qPCR	NPSG	HNP treatment; 45 m	*This study*
UCYN-C	2.1	qPCR	NPSG	C treatment; 45 m	*This study*
UCYN-C	2.2	qPCR	Southwest Pacific	P treatment	Turk-Kubo et al., 2015
*Richelia* in *Rhizosolenia* (Het-1)	1.3	qPCR	Southwest Pacific	P treatment	Turk-Kubo et al., 2015
*Richelia* in *Rhizosolenia* (Het-1)	1.7	qPCR	NPSG	LNP conditions; 45 m	*This study*
*Richelia* in *Rhizosolenia* (Het-1)	1.7	qPCR	NPSG	HNP treatment; 45 m	*This study*
*Richelia* in *Rhizosolenia* (Het-1)	1.4	qPCR	NPSG	C treatment; 45 m	*This study*
*Richelia* in *Hemiaulus* (Het-2)	2.2	qPCR	Southwest Pacific	P treatment	Turk-Kubo et al., 2015
*Richelia* in *Hemiaulus* (Het-2)	0.2	qPCR	NPSG	LNP treatment; 25 m	*This study*
*Richelia* in *Hemiaulus* (Het-2)	0.6	^15^N	North Pacific	*In situ*	Foster et al., 2011
*Calothrix* in *Chaetoceros* (Het-3)	1.1	qPCR	Southwest Pacific	P treatment	Turk-Kubo et al., 2015
*Trichodesmium*	0.1	^13^C	North Atlantic	*In situ*	Martinez-Perez et al., 2016
**Selected growth rates for cultivated isolates**					
*Cyanothece.* sp ATCC 51142	1.92	Culture	na	na	Vu et al., 2012
UCYN-C TW3	0.84	Culture	na	na	Taniuchi et al., 2012
*Cyanothece. sp* ATCC 51142	2.4	Culture	na	na	Reddy et al., 1993
*Crocosphaera watsonii* WH8501	0.49	Culture	na	na	Goebel et al., 2008
*Trichodesmium erythraeum* IMS101	0.51	Culture	na	na	Goebel et al., 2008
*Richelia* in *Rhizosolenia*	0.67	Culture	na	na	Villareal, 1989;Villareal, 1990


*Maximum rates from each study are reported and negative rates (when reported) are not included. NPSG, North Pacific Subtropical Gyre; ^*13*^C, measuring the incorporation of ^*13*^CO_*2*_ using nanoSIMS; qPCR, quantitative PCR; FCM, flow cytometry. ^*a*^Assumed by the authors of this study to be UCYN-A1 based on size and sample location. ^*b*^Assumed by the authors of this study to be UCYN-A3 based on size and sample location. ^*c*^Estimated from Figure 3 in Martinez-Perez et al. (2016).*

The maximum net growth rate measured for UCYN-A1 in this study, 1.3 d^-1^, is higher than reported in the North Atlantic (0.19 d^-1^; Moisander et al., 2011) and in the South Pacific (0.73 d^-1^; Turk-Kubo et al., 2015) (**Table 2**). These previously reported net growth rates were from experimental manipulations of surface waters amended with P [or P and P + Fe, in the case of Moisander et al. (2011)], conditions which are generally thought to favor diazotrophs. Interestingly, net growth rates of UCYN-A1 were uniformly high in samples from 45 m, independent of treatment (1.3 d^-1^), even though peak abundances of UCYN-A1 were measured at 25 m (**Figure 1**).

Maximum net growth rates for UCYN-B (1.5 d^-1^), UCYN-C (2.7 d^-1^), Het-1 (1.7 d^-1^) and Het-2 (0.2 d^-1^) were comparable to rates measured in a P-perturbation experiment in the New Caledonia lagoon (Turk-Kubo et al., 2015). As with UCYN-A1, maximum net growth rates were measured exclusively in the samples collected at 45 m, and were high across all treatments. This was not observed in the growth rates for *Prochlorococcus, Synechococcus* or the PPEs (**Figure 2**). These findings imply that all diazotrophic taxa were either growing at optimal maximum rates at the start of the incubation, and maintained these rates throughout the 48 h incubation, or that the combination of light, temperature and Fe was optimal for all taxa at this depth (10 nM Fe was added to all incubation bottles, including controls). These results are surprising given that peak abundances for all diazotrophs were measured at 25 m depth, as well as the physiological differences among diazotrophs and the evidence that there is niche partitioning throughout the water column (Moisander et al., 2010). Results from the grazing rate experiments clearly show that grazing pressure increases with depth, which may explain why peak abundances were found at 25 m, but the highest net growth rates were found at 45 m. Preliminary growth rate experiments conducted in July 2015 yielded very similar results, with net growth rates of the most abundant diazotrophs being high in control, HNP and LNP treatments at a single depth (see **Supplementary Figure S1**). Further research, and perhaps different tools for measuring growth rates in natural populations, are needed to determine the environmental factors behind this observation.

There have been relatively few measurements of *in situ* diazotroph growth rates, but when reported, they are often from nutrient perturbation experiments, where nutrient limitation may have been alleviated (**Table 2**). Due to this, along with differences in experimental design and measurement of growth rates, it is difficult to compare rates across studies, and between *in situ* and culture-based studies. However, results obtained in this and other recent studies together indicate that diazotrophs are capable of high net growth rates *in situ* over relatively short time periods, even in the presence of competition and mortality (grazing/viral lysis), which underscores the gaps in our understanding of their physiology and cellular requirements. In global ecological models, diazotroph growth rates are typically parameterized using ½ the maximum growth rate of a comparably sized non-diazotroph, to compensate for the energetic costs of fixing N_2_ (Dutkiewicz et al., 2009). Direct comparisons between large diazotrophs (*Trichodesmium*, DDAs) and their non-diazotrophic counterparts cannot be drawn from this study, but growth rates measured for the smaller, non-symbiotic diazotrophs (*Crocosphaera* and UCYN-C) are comparable to, and in many cases higher, than rates measured for *Synechococcus* (**Figure 2**).

### Diazotrophs Can Grow in Nitrate-Replete Conditions

Diazotrophs are predicted to be unable to coexist with non-diazotrophs at high N:P ratios under equilibrium conditions when Fe is not limiting (Schade et al., 2005). This is based on resource ratio theory (Dutkiewicz et al., 2009 and references therein), which uses basic physiological requirements for a specific phytoplankton taxa to define the minimum concentration of a given nutrient needed for survival. Thus, under the conditions in the HNP treatments of this study, non-diazotrophs would be expected to outcompete diazotrophs for available P (0.11 ± 0.01 μM at the beginning of the experiment). However, findings from this study indicate that diazotrophic taxa can compete for nutrients on relatively short temporal scales (48 h) under conditions thought to favor “velocity adapted” organisms capable of responding quickly to pulses of nutrients, such as diatoms (Sommer, 1984).

These findings may be explained in part due to growth possibly being uncoupled from the energetically demanding process of N_2_ fixation in HNP conditions, as some taxa can assimilate nitrate directly from the environment for growth (Agawin et al., 2007; Großkopf and LaRoche, 2012), or live in symbiosis with eukaryotic phytoplankton (diatoms, haptophytes) presumed to assimilate nitrate. The accessibility of a fixed N source has been shown to be a requirement for the initiation of N_2_ fixation in free-living soil diazotrophs, given the upfront investment a cell must make to generate N-containing cellular components (Norman and Friesen, 2017), thus the ability to acquire and utilize diverse sources of DIN and DON may also be a critical strategy for marine diazotrophs.

Further research is needed to verify the N source(s) used for growth by each diazotroph taxa under these conditions, and whether N_2_ fixation continues in nitrate-replete conditions. We cannot speculate about whether growth in these experiments was supported by increased N_2_ fixation or nitrate. However, there is growing evidence that N_2_ fixation *in situ* may not be as sensitive to DIN as previously thought (Knapp, 2012), especially when P concentrations are not limiting (Knapp et al., 2012). Meyer et al. (2016) recently reported that N_2_ fixation rates remained reasonably constant over an 8-day period and a broad range of nitrate concentrations in the Eastern Tropical North Atlantic, indicating that the presumably DDA-dominated diazotroph community (Het-1) could fix N_2_ in N replete conditions. Active N_2_ fixation has been measured in a variety of N-replete environments including high latitude Arctic waters (Shiozaki et al., 2017, 2018), and upwelling regions (Sohm et al., 2011a; Moreira-Coello et al., 2017).

UCYN-A1 lacks the genetic ability to directly assimilate nitrate (Tripp et al., 2010), so the observation that UCYN-A1 grows in the presence of nitrate deeper in the water column implies that when nitrate is available to the host, the symbiont either continues to fix N_2_ or obtains reduced N from its host to support growth. Although there have been several recent studies that have measured the response of UCYN-A1 to nutrient amendments (Moisander et al., 2011; Turk-Kubo et al., 2012; Krupke et al., 2015), it has been uncommon to include N species among the suite of nutrients added, since N_2_ fixation is understood to be either P or Fe limited (or P/Fe co-limited) in oligotrophic waters (Mills et al., 2004; Moore et al., 2009). However, two recent studies conducted in the Subtropical North Atlantic did include N addition as a treatment. Langlois et al. (2012) reported increased UCYN-A *nifH*-based abundances (ca. an order of magnitude) after the addition of ammonium nitrate to surface waters in one of the three experiments conducted in this region, indicating that the association was able to grow in N-replete conditions. However, despite greatly increased abundances of UCYN-A, no stimulation of bulk N_2_ fixation rates were measured in their N amended treatment. Without data on cell-specific N_2_ fixation rates it is unclear whether UCYN-A stopped fixing N_2_ in this incubation. However, Krupke et al. (2015) reported no reduction in cell-specific N_2_ fixation rates or *nifH* transcript production after the addition of 2 μM ammonium nitrate to UCYN-A populations in the North Atlantic suggesting that UCYN-A continues to fix N_2_ even when reduced N is available to the association.

It has been speculated that UCYN-A1 may reach peak abundances in oligotrophic waters with elevated nutrient conditions coinciding with entrainment of nutrient-rich waters from vertical mixing in the South Pacific Ocean (Moisander et al., 2010). At Station ALOHA, UCYN-A1 abundances peak in spring months when light intensity increases and the mixed layer begins to shoal, entraining nutrient-rich waters into the sunlit surface waters. However, it is unclear which component(s) of the deep nutrient pool are responsible for leading to peak UCYN-A abundances. Furthermore, Henke et al. (2018) found that UCYN-A1 peak abundances coincided with slightly elevated *in situ* nitrate concentrations in a time series conducted in the New Caledonia Lagoon. Results from this study may provide some insight into these observations, given that UCYN-A1 growth rates responded positively to the introduction of nitrate deeper in the water column. It is unclear why this was not the case for the samples collected at shallower depths, where more light energy was available for the prymnesiophyte host, and where the PPE population did have increased growth rates in the HNP treatment (**Figure 2A**), but active grazing on UCYN-A1 in surface waters may have depressed net growth rates. These findings underscore the likely tradeoff between conditions optimal for photosynthesis, N_2_ fixation and availability of nutrients, and provide valuable insight to this enigmatic symbiosis which may help explain its niche preferences for deeper waters (closer to the nutricline than other diazotrophs), and occurrences in colder, sometimes N-replete environments.

UCYN-B and UCYN-C, unicellular diazotrophs that may be free-living or living in aggregates (Sohm et al., 2011b; Bonnet et al., 2016), had similar responses across treatments and depths in the present study. As with UCYN-A1, their growth rates were stimulated by HNP conditions, but only at the surface (25 m), implying that light or a nutrient other than N was limiting growth in the HNP treatments deeper in the water column. Microzooplankton grazing pressure on UCYN-C was greatest near the DCM, and it is possible that the same may be true for UCYN-B. Positive growth rates in HNP conditions for UCYN-B are not unexpected given that *Crocosphaera* growth and N_2_ fixation are insensitive to nitrate concentrations in culture (Großkopf and LaRoche, 2012). The same is assumed to be true for UCYN-C in the NPSG, as *Cyanothece* isolates belonging to this group can grow on nitrate (Agawin et al., 2007). However, the qPCR assay used in this study does target a cluster that contains uncultivated marine sequence types, as well as the endosymbiont of the freshwater diatom *Rhopalodia gibba*. Therefore, without knowing with better certainty the source organism in the NPSG, the ability of the organisms targeted with the UCYN-C assay to assimilate nitrate remains speculative.

Stimulated growth rates in LNP treatments, conditions expected to favor diazotrophs due to N-limitation, were only measured for Het-2 at 25 m and UCYN-C and Het-1 at 45 m (**Figures 3O,J,N**). Notably, Het-2 was found only at the surface and did not have positive net growth rates in HNP treatments, unlike Het-1 (45 m). Both Het-1 and Het-2 lack the genetic capability to assimilate nitrate (Hilton et al., 2013; Hilton, 2014). The observation that Het-2 does not increase in abundance when its host is in an environment with replete nitrate availability, implies a different ecological strategy than the Het-1 symbiosis. Results from these incubations suggest that the Het-2 symbiosis has a competitive advantage in surface waters under LNP conditions in the NPSG.

### Microzooplankton Actively Graze on Diazotrophs

Very little is known about mortality processes for diazotrophs in the oligotrophic marine environment. To the best of our knowledge, this is the first report of the measurement of *in situ* microzooplankton grazing on UCYN-A1 and UCYN-C. Interestingly, these data suggest that the rate of microzooplankton grazing differed depending on the location in the water column and the grazed diazotroph taxa (**Figure 6**). UCYN-A1 experienced significant grazing pressure at the surface (GR2; *m* = 0.20 d^-1^) that exceeded measured growth rates. UCYN-C was not grazed at the surface, yet UCYN-A1 and UCYN-C were present at similar abundances at this depth (**Figure 1**). These findings imply that the grazer assemblage preyed preferentially on UCYN-A1 in surface waters. Different mortality rates measured for co-occurring photosynthetic populations (*Prochlorococcus, Synechococcus*, and PPEs) have been previously reported (Chen et al., 2009), and this situation appears to also be true for diazotrophs. Numerous factors contribute to preferential feeding of microzooplankton including prey size, cell surface properties, viral infection, nutrient composition, and the release of dissolved substances (Jürgens and Massana, 2008; Chrzanowski and Foster, 2014). However, cell size is generally a dominant factor determining the suitability of prey to microzooplankton consumers. UCYN-A1 lives in association with a prymnesiophyte, while UCYN-C occurs as free-living solitary cells. Thus, we speculate that differences in the size of the host-symbiont association of UCYN-A1 and the solitary cells of UCYN-C may explain the preferential grazing by the consumer assemblages on UCYN-A1 in surface waters. Size dependent grazing relationships would implicate microzooplanktonic protists (20–200 μm in size) as their consumers. This size category is dominated by ciliates, dinoflagellates and a variety of rhizarian taxa at Station ALOHA (Pasulka et al., 2013; Hu et al., unpublished data from the present study).

Interestingly, deeper in the water column, both diazotrophs were grazed at substantial rates (**Figure 6**), and there was general agreement between depth trends in the chl *a*-based grazing rates, which provide bulk microzooplankton grazing rates on the phytoplankton community, and qPCR-based grazing rates on diazotrophic taxa (**Table 2** and **Figure 4**). The mortality rate for UCYN-C at 125 m was the highest measured (1.75 d^-1^), and greater than both intrinsic and enriched growth rates for this cyanobacterial group. Additionally, in contrast to results from surface waters, the grazing rate on UCYN-A1 (1.00 d^-1^) was lower than the grazing rate for UCYN-C at this depth, and was also lower than its intrinsic growth rate (1.36 d^-1^), indicating that grazing pressure was not controlling UCYN-A1 abundances deeper in the water column. It is not clear if these depth-related differences in grazing pressure reflected differences in the feeding activities of the grazer community between the two depths, differences in the taxonomic composition of the consumer community, or both. General depression of grazing activity near the ocean surface might be inferred from the lower mortality rates observed for chl *a*-based measurements and the results for UCYN-C, but mortality of UCYN-A1 in surface waters was substantial in most cases. Moreover, previous work at station ALOHA (Pasulka et al., 2013) and molecular diversity studies conducted during the present study (Hu et al., unpublished; Connell et al., unpublished) have documented substantive changes in absolute and relative abundances of the various consumer taxa between surface waters and the lower euphotic zone (i.e., commensurate with the depths sampling in this study). Therefore, taxonomic composition and feeding behavior may both contribute to the depth-related differences in cyanobacterial mortality rates observed in this study.

More measurements are needed at finer depth resolution throughout the photic zone, and in different seasons (typically characterized by different dominant diazotrophic taxa) to better constrain microzooplankton grazing pressure on diazotroph populations, and to identify their consumers. However, our experiments verify that it is possible to use the dilution method technique to investigate mortality of diazotrophic taxa, when their abundances are high enough to remain above detection limits when diluted.

## Conclusion

Diazotroph growth and mortality rates are critical variables for global ecosystem models. The high energetic cost associated with the ability to fix N_2_ is typically offset in model parameterizations by assuming lower growth rates of diazotrophs relative to non-diazotrophic counterparts, as well as higher requirements for intracellular N:P and Fe:P ratios (e.g., Monteiro et al., 2010, 2011). Collectively, these assumptions are based on the tradeoffs of an organism which is never N-limited and the high energetic cost of N_2_ fixation. However, these assumptions are based primarily on *Trichodesmium* data and observations, and are validated by very few *in situ* measurements, particularly for unicellular diazotrophs. Furthermore, estimates of grazing on diazotrophs have been based on a simple interaction between predator and prey based on size (Monteiro et al., 2010) and sometimes palatability (Dutkiewicz et al., 2009). For these reasons, direct measurements of growth rates and microzooplankton grazing rates on diazotrophs are valuable, especially considering that for many of the important diazotroph groups, we are unable to define these terms using culture-based experiments.

The *in situ* growth rate experiment (G1) yielded several surprising results. In general, *in situ* net growth rates measured were high, often comparable to or greater than culture-based measurements (when comparisons can be made). LNP treatments, which were anticipated to provide diazotrophs with a competitive advantage, were not always the favorable conditions for growth at a given depth. Net growth rates for most diazotroph taxa appeared to be stimulated in the HNP treatments. More research is needed to determine whether the N needed for growth under these conditions was supported by assimilation of nitrate, or N_2_ fixation, acknowledging that the answer to this question is likely taxa-specific. However, these results suggest that the availability of DIN may be favorable on short time scales for natural populations of diazotrophs.

This is the first study to directly measure microzooplankton grazing rates on UCYN-A1 and UCYN-C. Future studies should focus on identifying the potential consumers of these minute diazotrophs, as well as using dilution grazing experiments to constrain mortality at higher spatial and temporal resolution. However, together with previously reported observations of copepod grazing on diazotrophs (O’Neil et al., 1996; O’Neil, 1998; Hunt et al., 2016; Conroy et al., 2017), these findings provide further indication that diazotroph derived N is, in some cases, directly transferred to higher trophic levels through grazing.

In summary, the high *in situ* net growth rates reported here, the competitive ability of some taxa in N-replete conditions, and measurement of depth-dependent microzooplankton grazing on diazotrophs, provide new insight into their ecological roles, and indicate that current parameterizations for this functional group in global ecosystem models may need reevaluation.

## Author Contributions

KT-K, HF, and JZ designed the growth rate experiment. PC, DC, KT-K, and JZ designed the grazing experiments. MH, KT-K, PC, and DC executed the experiments at sea. KT-K processed all samples for molecular and flow cytometry analysis, with help from MH. PC and DC processed chlorophyll samples from the grazing experiments. KT-K analyzed the data sets, with input from PC and DC on grazing rate experimental data. KT-K wrote the manuscript with input from all authors.

## Conflict of Interest Statement

The authors declare that the research was conducted in the absence of any commercial or financial relationships that could be construed as a potential conflict of interest. The handling Editordeclared a past co-authorship with one of the authors KT-K.

## Footnotes

1 www.arb-silva.de/download/arb-files

2 http://www.ncbi.nlm.nih.gov/sra

3 http://hahana.soest.hawaii.edu/hot/

## Supplementary Material

FIGURE S1 Results from growth rate experiments conducted in the vicinity of Station ALOHA in July 2015. Experiments were conducted and samples were processed as described for G1, but with water from a single depth (25 m) incubated in deck board incubators with *in situ* simulated light and temperature. The incubation was conducted during a *Crocosphaera* bloom described in detail in Wilson et al., 2017, but other diazotroph taxa were present **(A)**. Differential responses in net growth rates to HNP and LNP conditions were not seen for any of the phototrophic phytoplankton groups **(B)**. Diazotroph growth rates for the dominant diazotrophs **(C–H)** were uniformly high across all treatments and the controls, similar to the results from G1 at 45 m.

TABLE S1 Growth rate data used in **Figure 5**.

TABLE S2 OTU table, which includes taxonomy strings for each OTU for 16S rRNA data from G1.

## References

[B1] Agawin N. S. R. Rabouille S. Veldhuis M. J. W. Servatius L. Hol S. Van Overzee H. M. J. (2007). Competition and facilitation between unicellular nitrogen-fixing cyanobacteria and non-nitrogen-fixing phytoplankton species. *Limnol. Oceanogr.* 52 2233–2248. 10.4319/lo.2007.52.5.2233

[B2] Barcelose Ramos J. Schulz K. G. Voss M. Narciso Á. Müller M. N. Reis F. V. (2017). Nutrient-specific responses of a phytoplankton community: a case study of the north Atlantic gyre, azores. *J. Plankton Res.* 39 744–761. 10.1093/plankt/fbx025

[B3] Benavides M. Shoemaker K. M. Moisander P. H. Niggemann J. Dittmar T. Duhamel S. (2018). Aphotic N2 fixation along an oligotrophic to ultraoligotrophic transect in the western tropical south Pacific ocean. *Biogeosciences* 15 3107–3119. 10.5194/bg-15-3107-2018

[B4] Bentzon-Tilia M. Traving S. J. Mantikci M. Knudsen-Leerbeck H. Hansen J. L. S. Markager S. (2015). Significant N2 fixation by heterotrophs, photoheterotrophs and heterocystous cyanobacteria in two temperate estuaries. *ISME J.* 9 273–285. 10.1038/ismej.2014.119 25026373 PMC4303622

[B5] Bombar D. Heller P. Sanchez-Baracaldo P. Carter B. J. Zehr J. P. (2014). Comparative genomics reveals surprising divergence of two closely related strains of uncultivated UCYN-A cyanobacteria. *ISME J.* 8 2530–2542. 10.1038/ismej.2014.167 25226029 PMC4260699

[B6] Bombar D. Paerl R. W. Riemann L. (2016). Marine non-cyanobacterial diazotrophs: moving beyond molecular detection. *Trends Microbiol.* 24 916–927. 10.1016/j.tim.2016.07.002 27476748

[B7] Bombar D. Turk-Kubo K. A. Robidart J. C. Carter B. J. Zehr J. P. (2013). Non-cyanobacterial *nifH* phylotypes in the North Pacific Subtropical Gyre detected by flow-cytometry cell sorting. *Environ. Microbiol. Rep.* 5 705–715. 10.1111/1758-2229.12070 24115621

[B8] Bonnet S. Berthelot H. Turk-Kubo K. Fawcett S. Rahav E. L’helguen S. (2016). Dynamics of N2 fixation and fate of diazotroph-derived nitrogen in a low-nutrient, low-chlorophyll ecosystem: results from the VAHINE mesocosm experiment (New Caledonia). *Biogeosciences* 13 2653–2673. 10.5194/bg-13-2653-2016

[B9] Böttjer D. Dore J. E. Karl D. M. Letelier R. M. Mahaffey C. Wilson S. T. (2017). Temporal variability of nitrogen fixation and particulate nitrogen export at station ALOHA. *Limnol. Oceanogr.* 62 200–216. 10.1002/lno.10386

[B10] Boyle E. A. Bergquist B. A. Kayser R. A. Mahowald N. (2005). Iron, manganese, and lead at hawaii ocean time-series station ALOHA: temporal variability and an intermediate water hydrothermal plume. *Geochim. Cosmochim. Acta* 69 933–952. 10.1016/j.gca.2004.07.034

[B11] Capone D. Zehr J. Paerl H. Bergman B. (1997). *Trichodesmium*, a globally significant marine cyanobacterium. *Science* 276 1221–1229 10.1126/science.276.5316.1221

[B12] Capone D. G. Burns J. A. Montoya J. P. Subramaniam A. Mahaffey C. Gunderson T. (2005). Nitrogen fixation by *Trichodesmium* spp: an important source of new nitrogen to the tropical and subtropical north Atlantic ocean. *Global Biogeochem. Cycles* 19:GB2024 10.1029/2004GB002331

[B13] Caporaso J. G. Kuczynski J. Stombaugh J. Bittinger K. Bushman F. D. Costello E. K. (2010). QIIME allows analysis of high-throughput community sequencing data. *Nat. Methods* 7 335–336. 10.1038/nmeth.f.303 20383131 PMC3156573

[B14] Carradec Q. Pelletier E. Da Silva C. Alberti A. Seeleuthner Y. Blanc-Mathieu R. (2018). A global ocean atlas of eukaryotic genes. *Nat. Commun.* 9:373. 10.1038/s41467-017-02342-1 29371626 PMC5785536

[B15] Chen B. Liu H. Landry M. R. Dai M. Huang B. Sune J. (2009). Close coupling between phytoplankton growth and microzooplankton grazing in the western south China sea. *Limnol. Oceanogr.* 54 1084–1097. 10.4319/lo.2009.54.4.1084

[B16] Chrzanowski T. H. Foster B. L. (2014). Prey element stoichiometry controls ecological fitness of the flagellate ochromonas danica. *Aquat. Microb. Ecol.* 71 257–269. 10.3354/ame01680

[B17] Church M. Jenkins B. Karl D. Zehr J. (2005a). Vertical distributions of nitrogen-fixing phylotypes at Stn ALOHA in the oligotrophic north Pacific Ocean. *Aquat. Microb. Ecol.* 38 3–14. 10.3354/ame038003

[B18] Church M. Short C. Jenkins B. Karl D. Zehr J. (2005b). Temporal patterns of nitrogenase gene (nifH) expression in the oligotrophic north pacific ocean. *Appl. Environ. Microbiol.* 71 5362–5370. 10.1128/AEM.71.9.5362-5370.2005 16151126 PMC1214674

[B19] Church M. J. Mahaffey C. Letelier R. M. Lukas R. Zehr J. P. Karl D. M. (2009). Physical forcing of nitrogen fixation and diazotroph community structure in the north Pacific subtropical gyre. *Global Biogeochem. Cycles* 23:GB2020 10.1029/2008GB003418

[B20] Conroy B. J. Steinberg D. K. Song B. Kalmbach A. Carpenter E. J. Foster R. A. (2017). Mesozooplankton graze on cyanobacteria in the amazon river plume and western tropical north Atlantic. *Front. Microbiol.* 8:1436. 10.3389/fmicb.2017.01436 28824569 PMC5540951

[B21] Dekaezemacker J. Bonnet S. (2011). Sensitivity of N2 fixation to combined nitrogen forms (NO_3_^-^ and NH_4_^-^) in two strains of the marine diazotroph *Crocosphaera watsonii* (Cyanobacteria). *Mar. Ecol. Prog. Ser.* 438 33–46. 10.3389/fmicb.2012.00374

[B22] Dore J. E. Brum J. R. Tupas L. M. Karl D. M. (2002). Seasonal and interannual variability in sources of nitrogen supporting export in the oligotrophic subtropical north Pacific Ocean. *Limnol. Oceanogr.* 47 1595–1607. 10.4319/lo.2002.47.6.1595

[B23] Dutkiewicz S. Follows M. J. Bragg J. G. (2009). Modeling the coupling of ocean ecology and biogeochemistry. *Global Biogeochem. Cycles* 23:GB4017 10.1029/2008GB003405

[B24] Edgar R. C. (2010). Search and clustering orders of magnitude faster than BLAST. *Bioinformatics* 26 2460–2461. 10.1093/bioinformatics/btq461 20709691

[B25] Eren A. M. Maignien L. Sul W. J. Murphy L. G. Grim S. L. Morrison H. G. (2013). Oligotyping: differentiating between closely related microbial taxa using 16S rRNA gene data. *Methods Ecol. Evol.* 4 1111–1119. 10.1111/2041-210X.12114 24358444 PMC3864673

[B26] Falcon L. Carpenter E. Cipriano F. Bergman B. Capone D. (2004). N2 fixation by unicellular bacterioplankton from the Atlantic and Pacific Oceans: phylogeny and in situ rates. *Appl. Environ. Microbiol.* 70 765–770. 10.1128/AEM.70.2.765-770.2004 14766553 PMC348867

[B27] Falkowski P. (1997). Evolution of the nitrogen cycle and its influence on the biological sequestration of CO2 in the ocean. *Nature* 387 272–275. 10.1038/387272a0

[B28] Farnelid H. Turk-Kubo K. Munoz-Marin M. D. Zehr J. P. (2016). New insights into the ecology of the globally significant uncultured nitrogen-fixing symbiont UCYN-A. *Aquat. Microb. Ecol.* 77 125–138. 10.3354/ame01794

[B29] Fernandez C. Farias L. Ulloa O. (2011). Nitrogen fixation in denitrified marine waters. *PLoS ONE* 6:e20539. 10.1371/journal.pone.0020539 21687726 PMC3110191

[B30] Follett C. L. White A. E. Wilson S. T. Follows M. J. (2018). Nitrogen fixation rates diagnosed from diurnal changes in elemental stoichiometry. *Limnol. Oceanogr.* 10.1002/lno.10815 [Epub ahead of print].

[B31] Fontanez K. M. Eppley J. M. Samo T. J. Karl D. M. Delong E. F. (2015). Microbial community structure and function on sinking particles in the north Pacific subtropical gyre. *Front. Microbiol.* 6:469. 10.3389/fmicb.2015.00469 26042105 PMC4436931

[B32] Foster R. A. Kuypers M. M. M. Vagner T. Paerl R. W. Musat N. Zehr J. P. (2011). Nitrogen fixation and transfer in open ocean diatom-cyanobacterial symbioses. *ISME J.* 5 1484–1493. 10.1038/ismej.2011.26 21451586 PMC3160684

[B33] Foster R. A. Subramaniam A. Mahaffey C. Carpenter E. J. Capone D. G. Zehr J. P. (2007). Influence of the Amazon River plume on distributions of free-living and symbiotic cyanobacteria in the western tropical north Atlantic ocean. *Limnol. Oceanogr.* 52 517–532. 10.4319/lo.2007.52.2.0517

[B34] Fu F. X. Bell P. R. F. (2003). Growth, N2 fixation and photosynthesis in a cyanobacterium, *Trichodesmium* sp., under Fe stress. *Biotechnol. Lett.* 25 645–649. 10.1023/A:1023068232375 12882160

[B35] Goebel N. L. Edwards C. A. Carter B. J. Achilles K. M. Church M. J. Zehr J. P. (2008). Growth and carbon content of three different sized diazotrophic cyanobacteria observed in the subtropical North Pacific. *J. Phycol.* 44 1212–1220. 10.1111/j.1529-8817.2008.00581 27041718

[B36] Goebel N. L. Turk K. A. Achilles K. M. Paerl R. W. Hewson I. Morrison A. E. (2010). Abundance and distribution of major groups of diazotrophic cyanobacteria and their potential contribution to N2 fixation in the tropical Atlantic Ocean. *Environ. Microbiol.* 12 3272–3289. 10.1111/j.1462-2920.2010.02303.x 20678117

[B37] Gradoville M. R. Bombar D. Crump B. C. Letelier R. M. Zehr J. P. White A. E. (2017). Diversity and activity of nitrogen-fixing communities across ocean basins. *Limnol. Oceanogr.* 62 1895–1909. 10.1002/lno.10542

[B38] Green S. J. Venkatramanan R. Naqib A. (2015). Deconstructing the polymerase chain reaction: understanding and correcting bias associated with primer degeneracies and primer-template mismatches. *PLoS ONE* 10:e0128122. 10.1371/journal.pone.0128122 25996930 PMC4440812

[B39] Großkopf T. LaRoche J. (2012). Direct and indirect costs of dinitrogen fixation in *Crocosphaera watsonii* WH8501 and possible implications for the nitrogen cycle. *Front. Microbiol.* 3:236. 10.3389/fmicb.2012.00236 22833737 PMC3401090

[B40] Hamersley M. R. Turk K. A. Leinweber A. Gruber N. Zehr J. P. Gunderson T. (2011). Nitrogen fixation within the water column associated with two hypoxic basins in the southern California bight. *Aquat. Microb. Ecol.* 63 193–205. 10.3354/ame01494

[B41] Henke B. A. Turk-Kubo K. A. Bonnet S. Zehr J. P. (2018). Distributions and Abundances of sublineages of the N2-Fixing cyanobacterium Candidatus *Atelocyanobacterium thalassa* (UCYN-A) in the new caledonian coral lagoon. *Front. Microbiol.* 9:554. 10.3389/fmicb.2018.00554 29674998 PMC5895702

[B42] Herlemann D. P. Labrenz M. Jürgens K. Bertilsson S. Waniek J. J. Andersson A. F. (2011). Transitions in bacterial communities along the 2000 km salinity gradient of the Baltic sea. *ISME J.* 5 1571–1579. 10.1038/ismej.2011.41 21472016 PMC3176514

[B43] Hilton J. A. (2014). *Ecology and Evolution of Diatom-Associated Cyanobacteria Through Genetic Analyses.* Santa Cruz, CA: University of California.

[B44] Hilton J. A. Foster R. A. Tripp H. J. Carter B. J. Zehr J. P. Villareal T. A. (2013). Genomic deletions disrupt nitrogen metabolism pathways of a cyanobacterial diatom symbiont. *Nat. Commun.* 4:1767. 10.1038/ncomms2748 23612308 PMC3667715

[B45] Holl C. M. Montoya J. P. (2005). Interactions between nitrate uptake and nitrogen fixation in continuous cultures of the marine diazotroph, *Trichodesmium* (cyanobacteria). *J. Phycol.* 41 1178–1183. 10.1111/j.1529-8817.2005.00146.x

[B46] Hunt B. P. Conroy B. J. Foster R. A. (2016). Contribution and pathways of diazotroph-derived nitrogen to zooplankton during the VAHINE mesocosm experiment in the oligotrophic new caledonia lagoon. *Biogeosciences* 13 3131–3135. 10.5194/bg-13-3131-2016

[B47] Jürgens K. Massana R. (2008). “Protistan grazing on marine bacterioplankton,” in *Microbial Ecology of the Oceans*, 2nd Edn, ed. Gasol J. M. Kirchman D. L. (Hoboken, NY: John Wiley & Sons), 383–441. 10.1002/9780470281840.ch11

[B48] Karl D. M. Church M. J. Dore J. E. Letelier R. M. Mahaffey C. (2012). Predictable and efficient carbon sequestration in the north Pacific Ocean supported by symbiotic nitrogen fixation. *Proc. Natl. Acad. Sci.* 109 1842–1849. 10.1073/pnas.1120312109 22308450 PMC3277559

[B49] Knapp A. (2012). The sensitivity of marine N2 fixation to dissolved inorganic nitrogen. *Front. Microbiol.* 3:374. 10.3389/fmicb.2012.00374 23091472 PMC3476826

[B50] Knapp A. N. Dekaezemacker J. Bonnet S. Sohm J. A. Capone D. G. (2012). Sensitivity of *Trichodesmium erythraeum* and *Crocosphaera watsonii* abundance and N2 fixation rates to varying NO3- and PO43- concentrations in batch cultures. *Aquat. Microb. Ecol.* 66 223–236. 10.3354/ame01577

[B51] Krupke A. Mohr W. Laroche J. Fuchs B. M. Amann R. I. Kuypers M. M. (2015). The effect of nutrients on carbon and nitrogen fixation by the UCYN-A-haptophyte symbiosis. *ISME J.* 9 1635–1647. 10.1038/ismej.2014.253 25535939 PMC4478704

[B52] Landry M. Kirshtein J. Constantinou J. (1995). A refined dilution technique for measuring the community grazing impact of microzooplankton, with experimental tests in the central equatorial Pacific. *Mar. Ecol. Prog. Ser.* 120 53–63. 10.3354/meps120053

[B53] Landry M. R. Hassett R. P. (1982). Estimating the grazing impact of marine micro-zooplankton. *Mar. Biol.* 67 283–288. 10.1007/bf00397668

[B54] Langlois R. J. Mills M. M. Ridame C. Croot P. Laroche J. (2012). Diazotrophic bacteria respond to Saharan dust additions. *Mar. Ecol. Prog. Ser.* 470 1–14. 10.3354/meps10109

[B55] LaRoche J. Breitbarth E. (2005). Importance of the diazotrophs as a source of new nitrogen in the ocean. *J. Sea Res.* 53 67–91. 10.1038/ismej.2014.71 24813564 PMC4992080

[B56] Letelier R. M. Karl D. M. (1996). Role of *Trichodesmium* spp. in the productivity of the subtropical north Pacific Ocean. *Mar. Ecol. Prog. Ser.* 133 263–273. 10.3354/meps133263

[B57] Luo Y. W. Doney S. C. Anderson L. A. Benavides M. Berman-Frank I. Bode A. (2012). Database of diazotrophs in global ocean: abundance, biomass and nitrogen fixation rates. *Earth Syst. Sci. Data* 4 47–73. 10.5194/essd-4-47-2012

[B58] Martinez-Perez C. Mohr W. Loscher C. R. Dekaezemacker J. Littmann S. Yilmaz P. (2016). The small unicellular diazotrophic symbiont, UCYN-A, is a key player in the marine nitrogen cycle. *Nat. Microbiol.* 1:16163. 10.1038/nmicrobiol.2016.163 27617976

[B59] Meyer J. Löscher C. Neulinger S. Reichel A. Loginova A. Borchard C. (2016). Changing nutrient stoichiometry affects phytoplankton production, DOP accumulation and dinitrogen fixation–a mesocosm experiment in the eastern tropical north Atlantic. *Biogeosciences* 13 781–794. 10.5194/bg-13-781-2016

[B60] McMurdie P. J. Holmes S. (2013). phyloseq: an R package for reproducible interactive analysis and graphics of microbiome census data. *PloS ONE* 8:e61217. 10.1371/journal.pone.0061217 23630581 PMC3632530

[B61] Mills M. Ridame C. Davey M. Roche J. L. Geider R. (2004). Iron and phosphorus co-limit nitrogen fixation in the eastern tropical north Atlantic. *Nature* 429 292–294. 10.1038/nature02550 15152251

[B62] Moisander P. H. Beinart R. A. Hewson I. White A. E. Johnson K. S. Carlson C. A. (2010). Unicellular cyanobacterial distributions broaden the oceanic N2 fixation domain. *Science* 327 1512–1514. 10.1126/science.1185468 20185682

[B63] Moisander P. H. Beinart R. A. Voss M. Zehr J. P. (2008). Diversity and abundance of diazotrophic microorganisms in the south China sea during intermonsoon. *ISME J.* 2 954–967. 10.1038/ismej.2008.51 18528417

[B64] Moisander P. H. Benavides M. Bonnet S. Berman-Frank I. White A. E. Riemann L. (2017). Chasing after non-cyanobacterial nitrogen fixation in marine pelagic environments. *Front. Microbiol.* 8:1736. 10.3389/fmicb.2017.01736 28943875 PMC5596534

[B65] Moisander P. H. Zhang R. Boyle E. A. Hewson I. Montoya J. P. Zehr J. P. (2011). Analogous nutrient limitations in unicellular diazotrophs and Prochlorococcus in the south Pacific ocean. *ISME J.* 6 733–744. 10.1038/ismej.2011.152 22094348 PMC3309360

[B66] Monteiro F. M. Dutkiewicz S. Follows M. J. (2011). Biogeographical controls on the marine nitrogen fixers. *Global Biogeochem. Cycles* 25:GB2003 10.1029/2010GB003902

[B67] Monteiro F. M. Follows M. J. Dutkiewicz S. (2010). Distribution of diverse nitrogen fixers in the global ocean. *Global Biogeochem. Cycles* 24:GB3017 10.1029/2009GB003731

[B68] Montoya J. P. Holl C. M. Zehr J. P. Hansen A. Villareal T. A. Capone D. G. (2004). High rates of N2 fixation by unicellular diazotrophs in the oligotrophic Pacific ocean. *Nature* 430 1027–1031. 15329721 10.1038/nature02824

[B69] Moonsamy P. V. Williams T. Bonella P. Holcomb C. L. Hoglund B. N. Hillman G. (2013). High throughput HLA genotyping using 454 sequencing and the fluidigm access array system for simplified amplicon library preparation. *Tissue Antigens* 81 141–149. 10.1111/tan.12071 23398507

[B70] Moore C. M. Mills M. M. Achterberg E. P. Geider R. J. Laroche J. Lucas M. I. (2009). Large-scale distribution of Atlantic nitrogen fixation controlled by iron availability. *Nat. Geosci.* 2 867–871. 10.1038/NGEO667

[B71] Moreira-Coello V. Mouriño-Carballido B. Marañón E. Fernández-Carrera A. Bode A. Varela M. M. (2017). Biological N2 fixation in the upwelling region off NW iberia: magnitude, relevance, and players. *Front. Mar. Sci.* 4:303 10.3389/fmars.2017.00303

[B72] Mulholland M. R. Bernhardt P. W. Blanco-Garcia J. L. Mannino A. Hyde K. Mondragon E. (2012). Rates of dinitrogen fixation and the abundance of diazotrophs in north American coastal waters between cape hatteras and georges bank. *Limnol. Oceanogr.* 57 1067–1083. 10.4319/lo.2012.57.4.1067

[B73] Mulholland M. R. Capone D. G. (1999). Nitrogen fixation, uptake, and metabolism in natural and cultured populations of *Trichodesmium* spp. *Mar. Ecol. Prog. Ser.* 188 33–49. 10.3354/meps188033

[B74] Norman J. S. Friesen M. L. (2017). Complex N acquisition by soil diazotrophs: how the ability to release exoenzymes affects N fixation by terrestrial free-living diazotrophs. *ISME J.* 11 315–326. 10.1038/ismej.2016.127 27898052 PMC5270568

[B75] O’Neil J. M. (1998). The colonial cyanobacterium *Trichodesmium* as a physical and nutritional substrate for the harpacticoid Macrosetella gracilis. *J. Plankton Res.* 20 43–59. 10.1093/plankt/20.1.43

[B76] O’Neil J. M. Metzler P. M. Glibert P. M. (1996). Ingestion of 15N2-labelled *Trichodesmium* spp. and ammonium regeneration by the harpacticoid copepod *Macrosetella gracilis. Mar. Biol.* 125 89–96.

[B77] Pasulka A. L. Landry M. R. Taniguchi D. A. Taylor A. G. Church M. J. (2013). Temporal dynamics of phytoplankton and heterotrophic protists at station ALOHA. *Deep Sea Res. Part II Top. Stud. Oceanogr.* 93 44–57. 10.1016/j.dsr2.2013.01.007

[B78] Quast C. Pruesse E. Yilmaz P. Gerken J. Schweer T. Yarza P. (2013). The SILVA ribosomal RNA gene database project: improved data processing and web-based tools. *Nucleic Acids Res.* 41 D590–D596. 10.1093/nar/gks1219 23193283 PMC3531112

[B79] R Development Core Team (2012). *R: A Language and Environment for Statistical Computing. Foundation for Statistical Computing.* Available at: http://www.r-project.org/

[B80] Reddy K. Haskell J. Sherman D. Sherman L. (1993). Unicellular, aerobic nitrogen-fixing cyanobacteria of the genus *Cyanothece*. *J. Bacteriol.* 175 1284–1292. 10.1128/jb.175.5.1284-1292.1993 8444791 PMC193213

[B81] Sargent E. C. Hitchcock A. Johansson S. A. Langlois R. Moore C. M. Laroche J. (2016). Evidence for polyploidy in the globally important diazotroph *Trichodesmium*. *FEMS Microbiol. Lett.* 10.1093/femsle/fnw244 [Epub ahead of print]. 27797867

[B82] Scavotto R. E. Dziallas C. Bentzon-Tilia M. Riemann L. Moisander P. H. (2015). Nitrogen-fixing bacteria associated with copepods in coastal waters of the north Atlantic ocean. *Environ. Microbiol.* 17 3754–3765. 10.1111/1462-2920.12777 25655773

[B83] Schade J. D. Espeleta J. F. Klausmeier C. A. Mcgroddy M. E. Thomas S. A. Zhang L. (2005). A conceptual framework for ecosystem stoichiometry: balancing resource supply and demand. *Oikos* 109 40–51. 10.1111/j.0030-1299.2005.14050.x

[B84] Schmoker C. Hernández-León S. Calbet A. (2013). Microzooplankton grazing in the oceans: impacts, data variability, knowledge gaps and future directions. *J. Plankton Res.* 35 691–706. 10.1093/plankt/fbt023

[B85] Shilova I. Mills M. Robidart J. Turk-Kubo K. Björkman K. Kolber Z. (2017). Differential effects of nitrate, ammonium, and urea as N sources for microbial communities in the north Pacific ocean. *Limnol. Oceanogr.* 62 2550–2574. 10.1002/lno.10590

[B86] Shiozaki T. Bombar D. Riemann L. Hashihama F. Takeda S. Yamaguchi T. (2017). Basin scale variability of active diazotrophs and nitrogen fixation in the north pacific, from the tropics to the subarctic bering Sea. *Global Biogeochem. Cycles* 31 996–1009. 10.1002/2017GB005681

[B87] Shiozaki T. Fujiwara A. Ijichi M. Harada N. Nishino S. Nishi S. (2018). Diazotroph community structure and the role of nitrogen fixation in the nitrogen cycle in the chukchi sea (western Arctic Ocean). *Limnol. Oceanogr.* 10.1002/lno.10933 [Epub ahead of print].

[B88] Sohm J. A. Hilton J. A. Noble A. E. Zehr J. P. Saito M. A. Webb E. A. (2011a). Nitrogen fixation in the south Atlantic gyre and the Benguela upwelling system. *Geophys. Res. Lett.* 38:L16608 10.1029/2011GL048315

[B89] Sohm J. A. Subramaniam A. Gunderson T. E. Carpenter E. J. Capone D. G. (2011b). Nitrogen fixation by *Trichodesmium* spp. and unicellular diazotrophs in the north Pacific subtropical gyre. *J. Geophys. Res. Biogeosci.* 116:G03002 10.1029/2010JG001513

[B90] Sommer U. (1984). The Paradox of the plankton - fluctuations of phosphorus availability maintain diversity of phytoplankton in flow-through cultures. *Limnol. Oceanogr.* 29 633–636. 10.4319/lo.1984.29.3.0633

[B91] Sunagawa S. Coelho L. P. Chaffron S. Kultima J. R. Labadie K. Salazar G. (2015). Structure and function of the global ocean microbiome. *Science* 348:1261359. 10.1126/science.1261359 25999513

[B92] Taniuchi Y. Chen Y. -L. L. Chen H. -Y. Tsai M. -L. Ohki K. (2012). Isolation and characterization of the unicellular diazotrophic cyanobacterium Group C TW3 from the tropical western Pacific ocean. *Environ. Microbiol.* 14 641–654. 10.1111/j.1462-2920.2011.02606.x 21981769

[B93] Thompson A. Carter B. J. Turk-Kubo K. Malfatti F. Azam F. Zehr J. P. (2014). Genetic diversity of the unicellular nitrogen-fixing cyanobacteria UCYN-A and its prymnesiophyte host. *Environ. Microbiol.* 16 3238–3249. 10.1111/1462-2920.12490 24761991

[B94] Thompson A. W. Foster R. A. Krupke A. Carter B. J. Musat N. Vaulot D. (2012). Unicellular cyanobacterium symbiotic with a single-celled eukaryotic alga. *Science* 337 1546–1550. 22997339 10.1126/science.1222700

[B95] Tripp H. J. Bench S. R. Turk K. A. Foster R. A. Desany B. A. Niazi F. (2010). Metabolic streamlining in an open-ocean nitrogen-fixing cyanobacterium. *Nature* 464 90–94. 10.1038/nature08786 20173737

[B96] Turk-Kubo K. A. Achilles K. M. Serros T. R. Ochiai M. Montoya J. P. Zehr J. P. (2012). Nitrogenase (nifH) gene expression in diazotrophic cyanobacteria in the tropical north Atlantic in response to nutrient amendments. *Front. Microbiol.* 3:386. 10.3389/fmicb.2012.00386 23130017 PMC3487379

[B97] Turk-Kubo K. A. Farnelid H. M. Shilova I. N. Henke B. Zehr J. P. (2017). Distinct ecological niches of marine symbiotic N2-fixing cyanobacterium candidatus *atelocyanobacterium thalassa* sublineages. *J. Phycol.* 53 451–461. 10.1111/jpy.12505 27992651

[B98] Turk-Kubo K. A. Frank I. E. Hogan M. E. Desnues A. Bonnet S. Zehr J. P. (2015). Diazotroph community succession during the VAHINE mesocosm experiment (New Caledonia lagoon). *Biogeosciences* 12 7435–7452. 10.5194/bg-12-7435-2015

[B99] Turk-Kubo K. A. Karamchandani M. Capone D. G. Zehr J. P. (2014). The paradox of marine heterotrophic nitrogen fixation: abundances of heterotrophic diazotrophs do not account for nitrogen fixation rates in the eastern tropical south Pacific. *Environ. Microbiol.* 16 3095–3114. 10.1111/1462-2920.12346 24286454

[B100] Villareal T. A. (1989). *Ecology of Oceanic Diatoms: Investigations of Symbioses, Suspension and Growth Dynamics of Selected Rhizosolenia Species.* Kingston, RI: University of Rhode Island.

[B101] Villareal T. A. (1990). Laboratory culture and preliminary characterization of the nitrogen-fixing Rhizosolenia-Richelia symbiosis. *Mar. Ecol.* 11 117–132. 10.1111/j.1439-0485.1990.tb00233.x

[B102] Villareal T.A. (1992). “Marine nitrogen-fixing diatom – cyanobacteria symbioses,” in *Marine Pelagic Cyanobacteria: Trichodesmium and other Diazotrophs*, eds Carpenter E. J. Capone D. G. Rueter J. G. (Dordrecht: Kluwer Academic Publishers), 163–175.

[B103] Vu T. T. Stolyar S. M. Pinchuk G. E. Hill E. A. Kucek L. A. Brown R. N. (2012). Genome-scale modeling of light-driven reductant partitioning and carbon fluxes in diazotrophic unicellular cyanobacterium *Cyanothece* sp. ATCC 51142. *PLoS Comput. Biol.* 8:e1002460. 10.1371/journal.pcbi.1002460 22529767 PMC3329150

[B104] Ward B. A. Dutkiewicz S. Moore C. M. Follows M. J. (2013). Iron, phosphorus, and nitrogen supply ratios define the biogeography of nitrogen fixation. *Limnol. Oceanogr.* 58 2059–2075. 10.4319/lo.2013.58.6.2059

[B105] Welschmeyer N. A. (1994). Fluorometric analysis of chlorophyll a in the presence of chlorophyll b and phaeopigments. *Limnol. Oceanogr.* 39 1985–1992. 10.4319/lo.1994.39.8.1985

[B106] Wilson S. T. Aylward F. O. Ribalet F. Barone B. Casey J. R. Connell P. E. (2017). Coordinated regulation of growth, activity and transcription in natural populations of the unicellular nitrogen-fixing cyanobacterium *Crocosphaera*. *Nat. Microbiol.* 2:17118. 10.1038/nmicrobiol.2017.118 28758990

[B107] Zehr J. P. Bombar D. (2015). “Marine nitrogen fixation: organisms, significance, enigmas, and future directions,” in *Biological Nitrogen Fixation*, ed. de Bruijn F. J. (Santa Cruz, CA: University of California), 855–872. 10.1002/9781119053095.ch84

[B108] Zehr J. P. Montoya J. P. Jenkins B. D. Hewson I. Mondragon E. Short C. M. (2007). Experiments linking nitrogenase gene expression to nitrogen fixation in the North Pacific subtropical gyre. *Limnol. Oceanogr.* 52 169–183. 10.4319/lo.2007.52.1.0169

[B109] Zhang J. Kobert K. Flouri T. Stamatakis A. (2014). PEAR: a fast and accurate illumina paired-end reAd mergeR. *Bioinformatics* 30 614–620. 10.1093/bioinformatics/btt593. 24142950 PMC3933873

