# Supplementary material for: In Situ Diazotroph Population Dynamics Under Different Resource Ratios in the North Pacific Subtropical Gyre
Source: Front Microbiol. 2018 Jul 25;9:1616. doi: 10.3389/fmicb.2018.01616 (PMC6068237; doi:10.3389/fmicb.2018.01616)
Supplement: FIGURE S1 — Results from growth rate experiments conducted in the vicinity of Station ALOHA in July 2015. Experiments were conducted and samples were processed as described for G1, but with water from a single depth (25 m) incubated in deck board incubators with in situ simulated light and temperature. The incubation was conducted during a Crocosphaera bloom described in detail in Wilson et al., 2017, but other diazotroph taxa were present (A). Differential responses in net growth rates to HNP and LNP conditions were not seen for any of the phototrophic phytoplankton groups (B). Diazotroph growth rates for the dominant diazotrophs (C–H) were uniformly high across all treatments and the controls, similar to the results from G1 at 45 m. [file Data_Sheet_1.PDF]

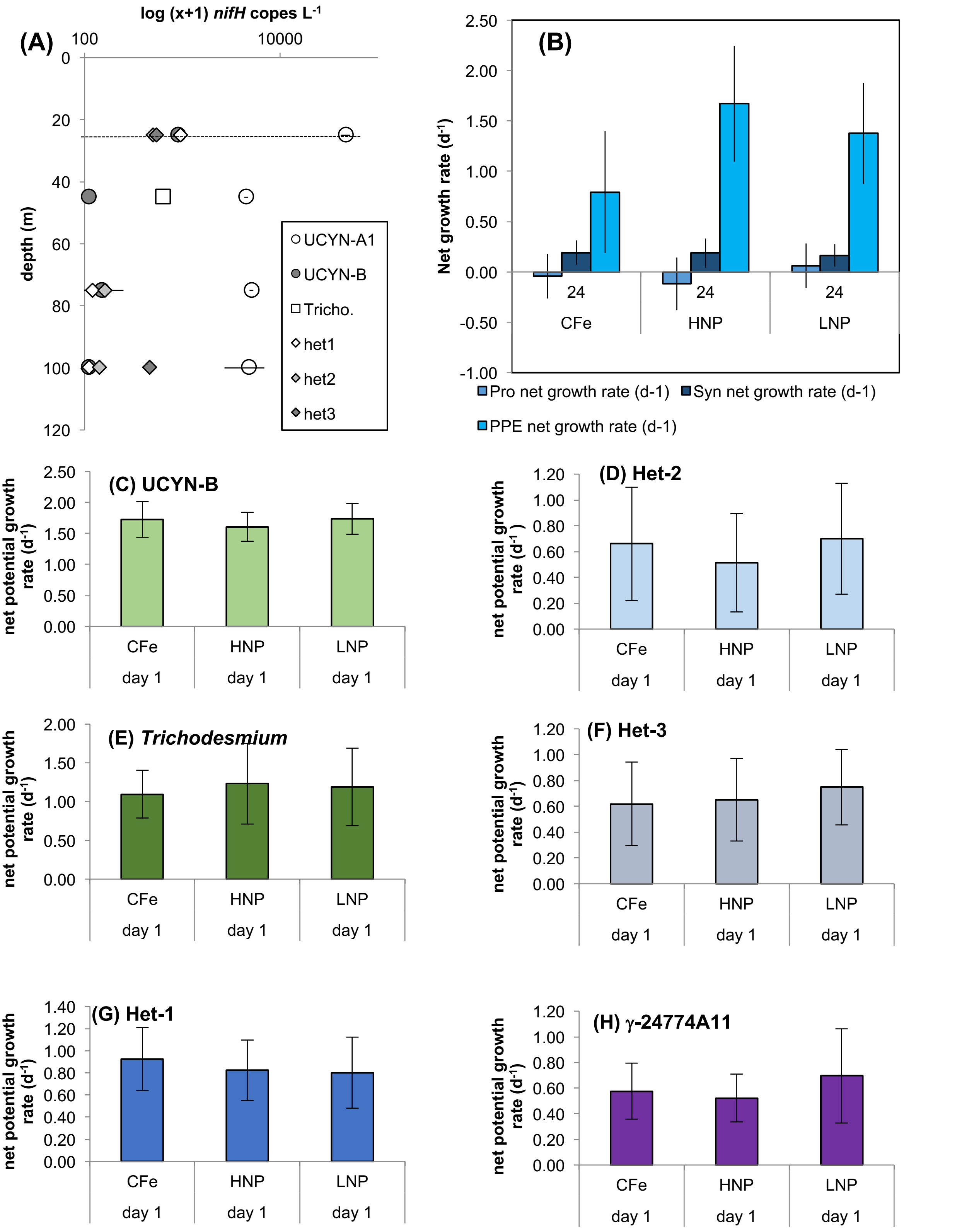

Supplemental Figure S1. Results from growth rate experiments conducted in the vicinity of Station ALOHA in July 2015. Experiments were conducted and samples were processed as described for G1, but with water from a single depth (25 m) incubated in deck board incubators with *in situ* simulated light and temperature. The incubation was conducted during a *Crocosphaera* bloom described in detail in Wilson et al., 2017, but other diazotroph taxa were present (A). Differential responses in net growth rates to HNP and LNP conditions were not seen for any of the phototrophic phytoplankton groups (B). Diazotroph growth rates for the dominant diazotrophs (C-H) were uniformly high across all treatments and the controls, similar to the results from G1 at 45 m.
